# Interaction between maternal immune activation and peripubertal stress in rats: impact on cocaine addiction-like behaviour, morphofunctional brain parameters and striatal transcriptome

**DOI:** 10.1038/s41398-023-02378-6

**Published:** 2023-03-08

**Authors:** Roberto Capellán, Javier Orihuel, Alberto Marcos, Marcos Ucha, Mario Moreno-Fernández, Marta Casquero-Veiga, María Luisa Soto-Montenegro, Manuel Desco, Marta Oteo-Vives, Marta Ibáñez-Moragues, Natalia Magro-Calvo, Miguel Ángel Morcillo, Emilio Ambrosio, Alejandro Higuera-Matas

**Affiliations:** 1 Department of Psychobiology, Faculty of Psychology, UNED, Madrid, Spain; 2 Instituto de Investigación Sanitaria Fundación Jiménez Díaz (IIS-FJD), Madrid, Spain; 3 Centro Nacional de Investigaciones Cardiovasculares (CNIC), Madrid, Spain; 4 Instituto de Investigación Sanitaria Gregorio Marañón, Madrid, Spain; 5 Grupo de Fisiopatología y Farmacología del Sistema Digestivo de la Universidad Rey Juan Carlos (NEUGUT), Madrid, Spain; 6 CIBER de Salud Mental (CIBERSAM), Madrid, Spain; 7 Departamento de Bioingeniería, Universidad Carlos III de Madrid, Madrid, Spain; 8 CIEMAT - Research Centre for Energy, Environment and Technology, Medical Applications of Ionizing Radiation Unit, Madrid, Spain

**Keywords:** Addiction, Schizophrenia

## Abstract

Substance use disorders are more prevalent in schizophrenia, but the causal links between both conditions remain unclear. Maternal immune activation (MIA) is associated with schizophrenia which may be triggered by stressful experiences during adolescence. Therefore, we used a double-hit rat model, combining MIA and peripubertal stress (PUS), to study cocaine addiction and the underlying neurobehavioural alterations. We injected lipopolysaccharide or saline on gestational days 15 and 16 to Sprague-Dawley dams. Their male offspring underwent five episodes of unpredictable stress every other day from postnatal day 28 to 38. When animals reached adulthood, we studied cocaine addiction-like behaviour, impulsivity, Pavlovian and instrumental conditioning, and several aspects of brain structure and function by MRI, PET and RNAseq. MIA facilitated the acquisition of cocaine self-administration and increased the motivation for the drug; however, PUS reduced cocaine intake, an effect that was reversed in MIA + PUS rats. We found concomitant brain alterations: MIA + PUS altered the structure and function of the dorsal striatum, increasing its volume and interfering with glutamatergic dynamics (PUS decreased the levels of NAA + NAAG but only in LPS animals) and modulated specific genes that could account for the restoration of cocaine intake such as the pentraxin family. On its own, PUS reduced hippocampal volume and hyperactivated the dorsal subiculum, also having a profound effect on the dorsal striatal transcriptome. However, these effects were obliterated when PUS occurred in animals with MIA experience. Our results describe an unprecedented interplay between MIA and stress on neurodevelopment and the susceptibility to cocaine addiction.

## Introduction

Substance use disorders are up to five times more prevalent in people with a diagnosis of schizophrenia than in the general population, affecting almost 50% of patients (this figure rises to 90% for nicotine use disorder) [1]. Moreover, substance use in people with schizophrenia is associated with increased morbidity and mortality, worsening the overall course of the disease, as suggested by increased rates of hospitalisation, lower treatment adherence, increased suicide rates, etc. [2]. In the specific case of cocaine, in a European sample, the risk of developing cocaine abuse was seven times higher in those with schizophrenia than in the general population [3].

In considering the neurodevelopmental origin of schizophrenia, maternal immune activation (MIA) models have arisen as a pivotal tool to investigate the comorbidity of neurodevelopmental disorders and addiction experimentally. MIA models recapitulate several aspects of the disease [4, 5]. They have been used to determine if behaviours reminiscent of specific aspects of addiction are increased in animals exposed to MIA (see Menne and Chesworth [1] and Ng et al. [6] for excellent reviews). In the case of cocaine, MIA induced by the synthetic viral RNA analogue polyinosinic:polycytidylic acid (poly I:C) increased cocaine-induced conditioned place preference (but decreased cocaine-induced locomotor activity) [7] and also potentiated cocaine cross-sensitisation after an amphetamine challenge [8]. However, when we tested if MIA induced by the bacterial endotoxin lipopolysaccharide (LPS) would potentiate cocaine self-administration, we found no evidence of such effect even in the presence of sensorimotor gating and cognitive deficits [9] or sensorimotor gating and immune alterations [10]. However, despite their translational validity, MIA models alone may not fully capture the complexity of the interactions between causative factors. More recently, these models are incorporating a second hit during adolescence, such as peripubertal stress of cannabinoid exposure, to increase their validity [11, 12].

In the present work, we aimed to expand our previous findings using a two-hit model that combines LPS-induced MIA and PUS to perform an extensive characterisation of the addiction-like behaviour (acquisition of cocaine self-administration, motivation for cocaine, compulsivity, loss of control over drug intake and incubation of seeking) [13, 14] and extensively study the neurodevelopmental alterations present in this model taking advantage of preclinical neuroimaging techniques (structural magnetic resonance imaging (MRI), diffusion tensor imaging (DTI), magnetic resonance spectroscopy (MRS) positron emission tomography (PET)) and next-generation sequencing (RNAseq).

We show unprecedented interactions between both hits in cocaine addiction-like behaviours and suggest that the hippocampal formation and the dorsolateral striatum could be significant brain loci mediating these interactions.

## Materials and methods

A complete description of the materials and methods followed can be found in the Supplementary Methods provided in the Supplementary Information.

### Experimental design

**Fig. 1:**
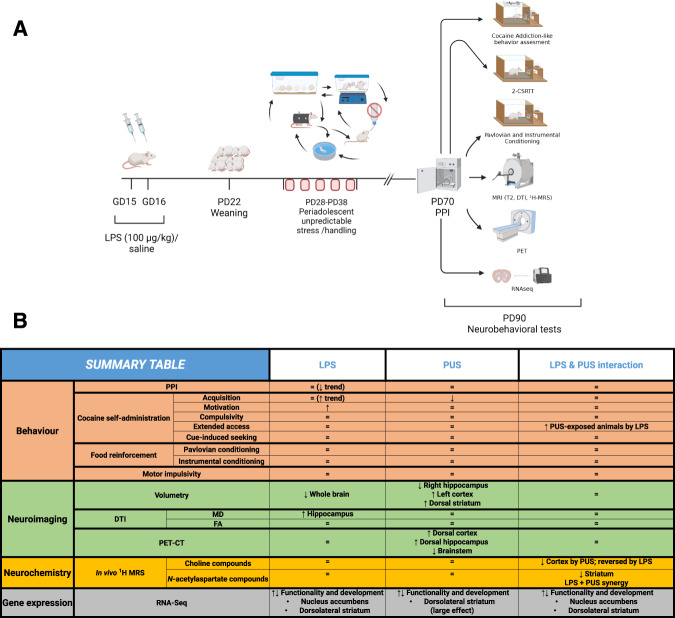
Experimental timeline and summary of the main findings. The figure shows an outline of the experimental design followed (**A**; created with Biorender.com) and the main findings obtained (**B**).

We performed five different experiments, carried out in independent batches of animals, aimed at testing: (1) cocaine addiction-like behaviour, (2) Pavlovian and instrumental conditioning, (3) impulsivity, (4) neuroimaging parameters and (5) transcriptomic alterations. In addition, all animals underwent a prepulse inhibition test (see Supplementary Information). Figure 1A shows an outline of the experimental design employed. For all the experiments described, dams were exposed to LPS or saline, and the male offspring underwent a series of stressful experiences during puberty or were handled to serve as controls (see below). Given that in a previous work we only found MIA-related effects in the male offspring [9], and that in a previous two-hit model using early LPS exposure and peripubertal stress, the authors found that females were almost unaffected [15], in this work we decided to test the males only. Therefore, our design resulted in four groups: SAL + NS (saline and no stress), SAL + S (saline and PUS), LPS + NS (animals exposed to MIA but not to PUS), and LPS + S (animals exposed to both hits). Sample sizes are indicated in the footnotes of the figures. Behavioural testing, brain imaging or sacrifice to obtain brain samples occurred upon reaching adulthood.

### Experimental animals

Experiments were performed on the male offspring of 14-week-old male and 12-week-old female Sprague-Dawley rats obtained from Charles River (France) following the EU Directive 2010/63/EU and approved by the Bioethics Committee of UNED and the Autonomous Community of Madrid (PROEX 078/18).

### Maternal immune activation

LPS (from *Escherichia coli* 0111: B4 [Sigma-Aldrich]) dissolved in 0.9% NaCl was intraperitoneally injected to pregnant rats at a dose of 100 mg/kg/ml on gestational days (GD) 15 and 16.

### Periadolescent unpredictable stress

Between PNDs 28 and 38, we exposed the male offspring to periadolescent unpredictable stress (PUS) consisting of (1) shaking; (2) immobilisation; (3) water deprivation; (4) forced swimming, and (5) constant changes of the home cage, each one applied every other day in a counterbalanced order. Non-stressed controls were handled by the same researcher on the same days as the stressed subjects.

### Experimental procedures

#### Cocaine self-administration

At PND80, animals underwent surgery to be implanted with a jugular vein catheter that allowed intravenous cocaine self-administration. On PND90, the cocaine self-administration (0.5 mg/kg i.v.) programme began with six different phases: (1) acquisition (12 2-h daily sessions under an FR1 schedule); (2) motivation test (6 2-h daily sessions under a progressive ratio programme); (3) stabilisation (3 sessions of identical conditions as those used in the acquisition phase); (4) compulsive seeking test (a single 1-h session under an FR3 schedule where the animals randomly received an infusion or a mild plant shock of 0.5 mA for 0.5 s); (5) extended access (10 6-h daily sessions under an FR1 schedule). At the end of this phase, a forced abstinence period was imposed to evaluate cue-induced relapse, leading to the sixth phase; (6) drug-seeking test (a 2-h session with no drug on days withdrawal days 1, 30, 60 and 100).

#### Pavlovian and instrumental conditioning

The Pavlovian learning protocol consisted of eight daily sessions where a CS predicted the delivery of a food pellet and another CS the absence of the reward. We measured the head entries to the food magazine after the presentation of each type of stimulus and calculated a ratio between them as a learning index. The instrumental learning protocol was performed after the Pavlovian training. It consisted of seven daily sessions where presses of an active lever led to the presentation of a food reward under different reinforcement schedules. An inactive lever was present but had no effect on food delivery.

#### Two-choice serial reaction time task (2-CSRTT)

We adapted the widely used 5-CSRTT [16] to measure sustained visual attention and waiting impulsivity. Rats had to withhold their responses on a specific lever until the appropriate stimulus appeared, marking the onset of the interval when responses on that marked lever were rewarded.

### Neuroimaging studies

#### Magnetic resonance imaging (MRI) and diffusion tensor imaging (DTI), and in vivo proton magnetic resonance spectroscopy (^1^H-MRS)

A 7.0 Tesla Pharmascan MRI scanner (Bruker, Germany) was used to acquire T2-weighted (T2-W) spin-echo anatomical images under isoflurane anaesthesia. Diffusion-weighted images were acquired with a spin-echo single-shot echo-planar imaging (EPI) pulse. Fractional anisotropy (FA), mean diffusivity (MD), trace, eigenvalues and eigenvector maps were calculated with a homemade software application written in Matlab (R2007a). Values were extracted from maps using regions of interest (ROIs) with Image J software.

Immediately after conducting the MRI and DTI analysis, we conducted an in vivo ^1^H MRS study. Given their involvement in schizophrenia and addiction we the chose the cerebral cortex and striatum as regions of interest: A Point-REsolved Spatially Spectroscopy (PRESS) was used, combined with a variable power radiofrequency (VAPOR) water suppression.

#### Positron emission tomography/computed tomography (PET-CT)

PET-CT studies were performed using a small-animal Argus PET/CT (SEDECAL, Madrid, Spain). PET studies (energy window 400–700 KeV and 45 min static acquisition) and CT (voltage 45 kV, current 150 μA, 8 shots, 360 projections and standard resolution) were performed 30 min after administration of 34.7 ± 3.02 MBq of [^18^F]-2-fluoro-2-deoxy-d-glucose (^18^F-FDG) via the tail vein in fasting rats anaesthetised with isoflurane. PET-CT image reconstruction was accomplished using a 2D-OSEM algorithm (16 subsets and 3 iterations) with random and scatter correction. Statistical Parametric Mapping (SPM) software was used for voxel-based analysis (http://www.fil.ion.ucl.ac.uk/spm/software/spm12/).

#### RNAseq

Once PND90 was reached, animals were sacrificed, and tissue from the nucleus accumbens (NAcc) and dorsolateral striatum was dissected. RNA extraction was performed using the RNeasy Mini Kit (Qiagen). Libraries were prepared according to the ‘NEBNext Ultra Directional RNA Library Prep kit for Illumina’ (New England Biolabs) instructions and sequenced using a ‘NextSeq™ 500 High Output Kit’ in a 1 × 75 single read sequencing run on a NextSeq500 sequencer. Differential expression analysis was carried out using the CUFFDIFF tool. We then used Metascape (https://metascape.org/gp/index.html#/main/step1) to analyse the enrichment in specific gene ontologies for each comparison.

### Statistical analysis

We used *Χ*^2^ tests and 2-way ANOVAs (followed by simple effects analyses after significant interactions) or non-parametric statistics (Kruskal–Wallis and Mann–Whitney tests) whenever the assumptions of ANOVA were not met. Data are presented as mean ± s.e.m. unless otherwise specified.

## Results

The main results obtained in all the studies are summarised in Fig. 1B.

### Cocaine-related behaviours

#### Acquisition patterns and initial cocaine self-administration

**Fig. 2:**
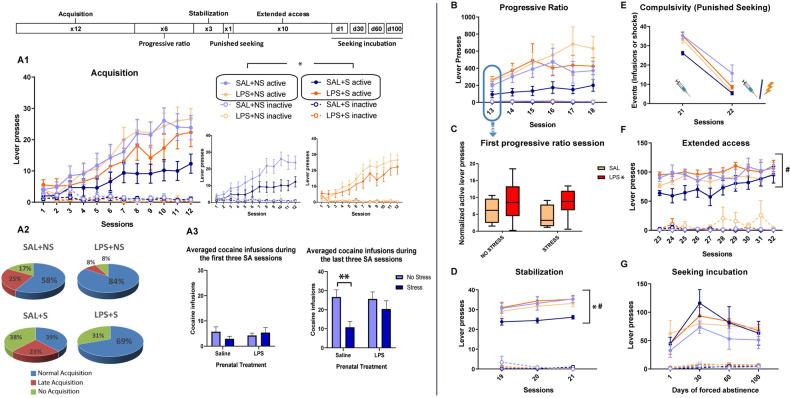
Cocaine self-administration programme. **A1** Active and inactive lever presses during the acquisition phase (SAL + NS: *n* = 13; SAL + S: *n* = 13; LPS + NS: *n* = 13; LPS + S: *n* = 15). All groups showed effective discrimination between active and inactive lever presses, as shown by the significant Lever factor (*F*_1,47_ = 72.233; *p* < 0.001; *η*^2^*p* = 0.606) and the Lever × Sessions factors interaction (*F*_1,47_ = 41.403; *p* *<*  0.001; *η*^2^*p* = 0.468). Notably, the Lever × MIA or the Lever × PUS interactions were not significant. In addition, no significant effects were found on inactive lever presses during this phase. **A2** The results of the categorical analysis showing the percentage of animals with regular, delayed, or absent cocaine self-administration acquisition. To carry out this qualitative analysis of the acquisition phase, animals were categorised as showing: “Regular acquisition” (animals that showed cocaine self-administration behaviour, i.e. more than five infusions over the last 3 days of acquisition), “Late acquisition” (animals that took more than 12 sessions to present a stable behaviour of cocaine self-administration) and “No-acquisition” (animals that by the end of the experiment never showed a cocaine self-administration behaviour). **A3** Number of cocaine infusions over the first or last three self-administration sessions. **B** Active and inactive lever presses during the progressive ratio phase (SAL + NS: *n* = 9; SAL + S: *n* = 8; LPS + NS: *n* = 12; LPS + S: *n* = 9). Discrimination between active and inactive lever presses was significant in all groups, as shown by the Lever factor (*F*_1,34_ = 40.674; *p* = 0.000; *η*^2^*p* = 0.610) and Lever × Sessions factors interaction (*F*_1,34_ = 5.276; *p* = 0.002; *η*^2^*p* = 0.169). **C** Normalised active lever presses in the first progressive ratio session (active lever presses in the first progressive ratio session/active lever presses in the last acquisition phase session). **D** Active and inactive lever presses during the stabilisation phase (SAL + NS: *n* = 9; SAL + S: *n* = 6; LPS + NS: n = 11; LPS + S: *n* = 8). Discrimination between active and inactive lever presses was significant in all groups, as shown by the Lever factor (*F*_1,29_ = 853.870; *p* < 0.001; *η*^2^*p* = 0.967) and Lever × Sessions factors interaction (*F*_1,29_ = 7.935; *p* = 0.002; *η*^2^*p* = 0.215). **E** Reduction in drug-seeking due to foot shock represented as a decrease in the events (infusions or shocks) registered during the compulsive-seeking session (SAL + NS: *n* = 7; SAL + S: *n* = 6; LPS + NS: *n* = 11; LPS + S: *n* = 8) regarding those registered in the last stabilisation session; **F** active and inactive lever presses during the extended access phase (SAL + NS: *n* = 8; SAL + S: *n* = 6; LPS + NS: *n* = 11; LPS + S: *n* = 8). Discrimination between active and inactive lever presses was significant in all groups by the Lever factor (*F*_1,23_ = 244.412; *p* < 0.001; *η*^2^*p* = 0.914). **G** Active and inactive lever presses during the seeking incubation phase (SAL + NS: *n* = 5; SAL + S: *n* = 5; LPS + NS: *n* = 10; LPS + S: *n* = 8). Discrimination between active and inactive lever presses was significant in all groups, as shown by the Lever factor (*F*_1,24_ = 81.650; *p* < 0.001; *η*^2^*p* = 0.773) and Lever × Sessions factors interaction (*F*_1,24_ = 9.199; *p* = 0.001; *η*^2^*p* = 0.277). Single asterisk (*) denotes a *p* < 0.05 while double asterisks (**) denotes a *p* < 0.01 difference compared to the stressed group among saline-exposed rats; # denotes a *p* < 0.05 difference compared to the respective saline group among PUS-exposed rats.

Our first approach to the data was to perform a categorical analysis of the acquisition patterns displayed by the rats. (Fig. 2 (A2)). Although there were no significant differences between groups in the overall analysis (*Χ*^2^_6_ = 9.553; *p* = 0.1450), we observed that the group of LPS-exposed animals (without stress) had a strong trend (*Χ*^2^_2_ = 5.887; *p* = 0.053) to have a higher proportion of rats displaying robust cocaine self-administration (greater “Normal acquisition” percentage). Within the stressed rats (not exposed to LPS), no significant differences were obtained (*Χ*^2^_6_ = 3.625; *p* = 0.163), suggesting that stressful experiences during periadolescence do not affect the percentage of individuals acquiring cocaine self-administration.

All rats progressively increased the number of lever presses during training (significant effects of the Sessions factor (*F*_1,47_ = 43.071; *p* = 0.0001; *η*^2^*p* = 0.478)); however, as seen in Fig. 2 (A1), PUS significantly reduced cocaine self-administration (*F*_1,47_ = 4.409; *p* = 0.041; *η*^2^*p* = 0.086), an effect that was more evident at the end of the training, as revealed by the significant Sessions × PUS interaction (*F*_1,47_ = 3.718; *p* = 0.007; *η*^2^*p* = 0.073). Interestingly, the effect of PUS was restricted to SAL-exposed animals, while LPS rats seemed to be resistant to the impact of PUS on cocaine self-administration acquisition (Fig. 2 (A3)).

Other critical parameters that indicate a tendency to develop compulsive cocaine intake, such as the number of lever presses during the time out period, the mean latency to the first lever press or the mean inter-response time, were not affected (data not shown).

For the analysis of the motivation to consume cocaine, we used a progressive ratio schedule. We first focused on the performance during the first session as it is devoid of the effects of the progressive behavioural adaptation to the schedule. Active lever press values in the first session were normalised against those of the last acquisition session to rule out a possible influence of the consumption pattern exhibited in the previous phase. Using this index, we observed that LPS-exposed animals showed greater motivation for cocaine consumption (*F*_1,34_ = 6.628; *p* = 0.015; *η*^2^*p* = 0.163) in the first session of the progressive ratio phase (day 13) (Fig. 2B, C). A significant effect of the Sessions factor was found in the active lever presses (*F*_1,34_ = 5.206; *p* = 0.002; *η*^2^*p* = 0.167) (Fig. 2B) and in the breaking points (*F*_1,34_ = 5.096; *p* = 0.002; *η*^2^*p* = 0.164) (Fig. S2) reached during the progressive ratio phase. However, no significant differences were obtained between the groups. Inactive lever presses were not significantly altered during this phase (Fig. 2B).

When animals were returned to FR1, we observed a progressive increase in active lever presses (*F*_1,29_ = 7.432; *p* = 0.001; *η*^2^*p* = 0.204) and significant differences between the groups (as revealed by the interaction between MIA and PUS (*F*_1,29_ = 7.316; *p* = 0.011; *η*^2^*p* = 0.201)). MIA reversed the reduction in cocaine self-administration induced by PUS (indeed, LPS-stressed rats self-administered more cocaine than SAL-stressed rats (*F*_1,29_ = 6.21; *p* = 0.018), and SAL-stressed rats displayed lower intake than SAL-non-stressed rats (*F*_1,29_ = 7.54; *p* = 0.010) (Fig. 2D)). No significant effects were found in inactive lever presses during this phase (Fig. 2D). Compulsive cocaine-seeking was not affected by MIA or PUS. Indeed, when we compared the drug infusions earned during the last session of the stabilisation phase with the number of infusions/shocks received in the compulsive seeking test, we observed that all rats reduced their drug-seeking (significant effect of the Sessions factor (*F*_1,28_ = 388.172; *p* = 0.000; *η*^2^*p* = 0.933), regardless of MIA, PUS or their combination (Fig. 2E). During the extended access phase, we observed significant effects of the Sessions factor (*F*_1,23_ = 4.945; *p* = 0.004; *η*^2^*p* = 0.177) and the interaction between MIA and PUS (*F*_1,23_ = 4.893; *p* = 0.037; *η*^2^*p* = 0.175). Prenatal LPS treatment normalised cocaine self-administration among stressed animals (*F*_1,23_ = 4.889; *p* = 0.035). No significant effects were found on inactive lever presses during this phase (Fig. 2F). When we analysed drug-seeking after different withdrawal times, we detected a significant effect of the Sessions factor (*F*_1,24_ = 10.989; *p* < 0.001; *η*^2^*p* = 0.314), suggestive of the incubation of seeking phenomenon. However, no significant differences were obtained between the groups (Fig. 2G).

To further understand the psychological processes mediating these results, we examined Pavlovian and instrumental conditioning (using food as a reinforcer). We found no differences, ruling out the involvement of such mechanisms in cocaine addiction-like behaviour data (see supplementary results and Fig. S3A, B). Moreover, impulsive behaviour, a critical endophenotype conferring vulnerability to cocaine addiction, was not affected either (Fig. S4).

### Neuroimaging studies

#### MRI-assisted volumetry

**Fig. 3:**
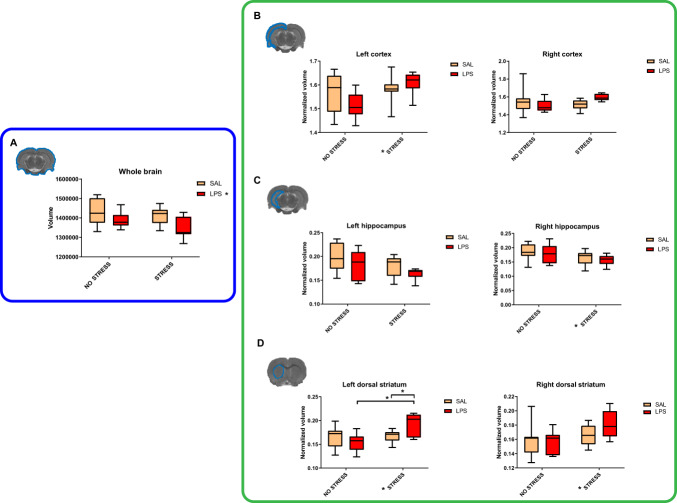
Brain regional volumetry. The figure shows the volume of the whole brain (**A**), left and right cortex (**B**), left and right hippocampus (**C**), and left and right dorsal striatum (**D**). The volumetric quantification of each brain structure was normalised to the volume of the corresponding MRI section. (SAL + NS: *n* = 8; SAL + S: *n* = 8; LPS + NS: *n* = 8; LPS + S: *n* = 7). Single asterisk (*) in a boxplot denotes a *p* < 0.05 difference among specific groups. Single asterisk (*) in the legend denotes a significant main effect (*p* < 0.05) of the maternal immune activation factor.

Having characterised cocaine addiction-like behaviour in this model, we decided to use MRI to search for brain alterations that could be underlying the behavioural patterns obtained. Whole-brain volume was reduced as a consequence of MIA (*F*_1,27_ = 6.520; *p* = 0.017; *η*^2^*p* = 0.195) (Fig. 3A). Regarding the interactions between MIA and PUS, we observed a significant effect in the volume of the left dorsal striatum (*F*_1,27_ = 6.105; *p* = 0.020; *η*^2^*p* = 0.184), whereby LPS-exposed stressed rats showed a significant increase in volume compared to non-stressed (*F*_1,27_ = 12.519; *p* = 0.001) and saline-exposed (*F*_1,27_ = 5.018; *p* = 0.034) controls (Fig. 3D). A MIA × PUS interaction was also evident in the right cortical volume (*F*_1,27_ = 4.484; *p* = 0.044; *η*^2^*p* = 0.025) (Fig. 3B). However, no significant differences were observed in subsequent simple effects analysis. On the other hand, PUS increased both the left cortical volume (*F*_1,27_ = 5.709; *p* = 0.024; *η*^2^*p* = 0.175) (Fig. 3B) and the dorsostriatal volume bilaterally (Right: *F*_1,27_ = 5.289; *p* = 0.029; *η*^2^*p* = 0.164/Left: *F*_1,27_ = 6.856; *p* = 0.014; *η*^2^*p* = 0.203) (Fig. 3D), but decreased the right hippocampus in the LPS animals (*F*_1,27_ = 4.417; *p* = 0.045; *η*^2^*p* = 0.141) (Fig. 3C).

No significant effects of MIA, PUS or their interaction were found in cerebellar, amygdalar and NAcc volumes (Fig. S5A–C), nor in the fourth ventricle, third ventricle, lateral ventricles, cerebral aqueduct, and total ventricular volumes (Fig. S6A–F).

#### DTI

**Fig. 4:**
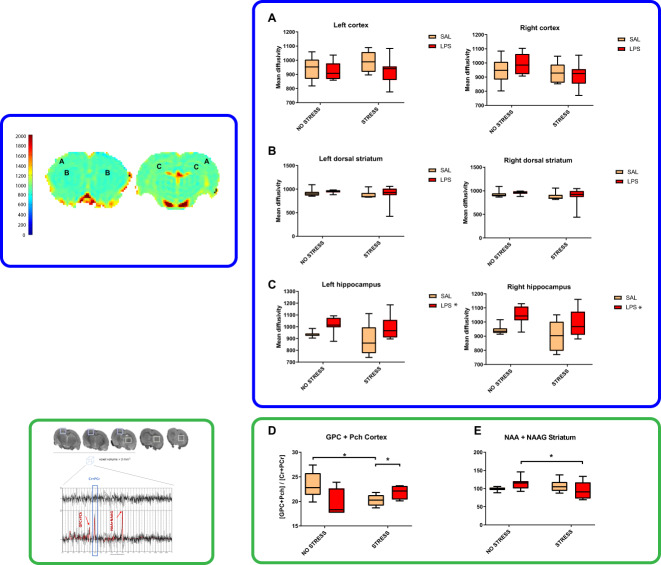
DTI: mean diffusivity measurements in specific brain areas and ^1^H-MRS: normalised [GPC + PCh] levels in the cortex and [NAA + NAAG] levels in the striatum. The figure shows mean diffusivity values in the cortex (**A**), dorsal striatum (**B**) and hippocampus (**C**) in both hemispheres. (SAL + NS: *n* = 8; SAL + S: *n* = 8; LPS + NS: *n* = 8; LPS + S: *n* = 8) and normalised [GPC + PCh] levels in the cortex (**D**) (SAL + NS: *n* = 6; SAL + S: *n* = 6; LPS + NS: *n* = 4; LPS + S: *n* = 4) and normalised [NAA + NAAG] levels in the striatum (**E**) (SAL + NS: *n* = 7; SAL + S: *n* = 8; LPS + NS: *n* = 8; LPS + S: *n* = 7). Single asterisk (*) in a boxplot denotes a *p* < 0.05 difference among specific groups.

After the volumetry analysis, we looked at tissue microarchitecture. MD was not different between groups in the cortex or the dorsal striatum (Fig. 4A, B). However, we observed that this parameter was significantly increased in the right (*U* = 60, *p* = 0.0177) and left (*U* = 58, *p* = 0.0143) hippocampi of LPS-exposed animals (Fig. 4C). No alterations in white matter integrity were observed in our analysis. Indeed, there were no changes in FA values in any of the structures mentioned (Fig. S7A–C) or in the main cerebral tracts (corpus callosum, internal capsule and anterior commissure) (Fig. S8A–C) as a consequence of MIA, PUS, or their interaction.

#### ^1^H-MRS

MRS only allows the examination of large structures, so we decided to examine brain regions with known involvement in schizophrenia and addiction. To this end, we selected the brain cortex and dorsal striatum. We obtained a MIA × PUS interaction in the levels of choline compounds (glycerophosphorylcholine + phosphorylcholine (GPC + PCh) peak) in the cortex (*F*_1,16_ = 6.940, *p* = 0.018, *η*^2^*p* = 0.303). While PUS reduced the relative amount of these metabolites (*F*_1,16_ = 6.246, *p* = 0.024), the combination with MIA reversed this effect and slightly increased the levels of this metabolite among stressed animals (*F*_1,16_ = 6.481, *p* = 0.022) (Fig. 4D).

With regard to the *N*-acetylaspartate + *N*-acetylaspartylglutamic acid (NAA + NAAG) peak levels, we also obtained a MIA × PUS in the striatum (*F*_1,26_ = 4.776, *p* = 0.038, *η*^2^*p* = 0.155) whereby PUS reduced the relative amount of these metabolites only in LPS-exposed animals (*F*_1,26_ = 4.495, *p* = 0.044) (Fig. 4E).

#### PET

**Fig. 5:**
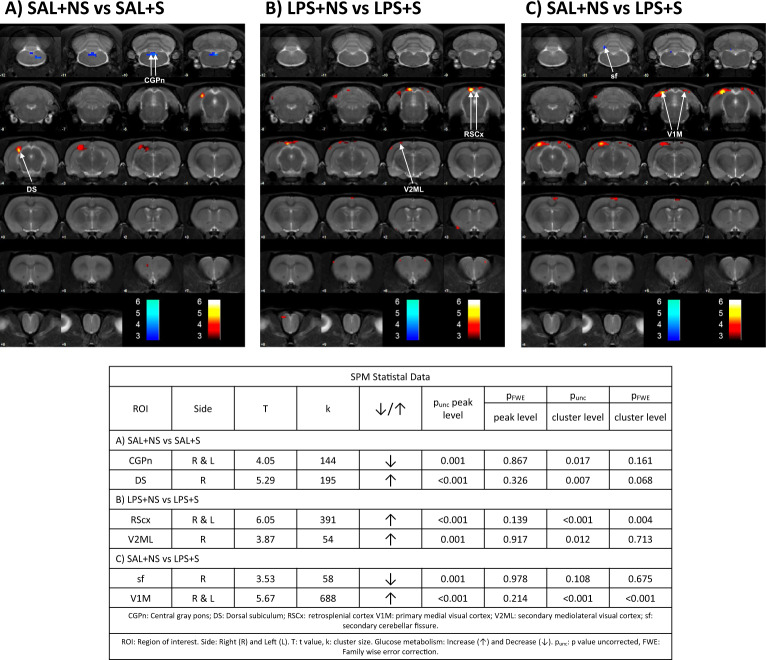
SPM analysis of [^18^F]-FDG for the metabolic activity. Coloured PET images: T-maps overlaid on an MRI template indicate reduced (blue) and increased (red) FDG uptake. The colour bars represent the *p* level of the voxel-wise comparisons of the brain metabolic activity. The figure shows the effects found in the SAL + S compared to SAL + NS rats (**A**), the effects found in the LPS + S compared to LPS + NS rats (**B**) and the effects found in LPS + S rats compared to SAL + NS animals (**C**). The statistical significance threshold between groups was set at *p* < 0.01 (uncorrected) and *k* > 50 voxels. CGPn central grey pons, DS dorsal subiculum, RSCx retrosplenial cortex, V1M primary medial visual cortex, V2ML secondary mediolateral visual cortex, sf secondary cerebellar fissure (SAL + NS: *n* = 5; SAL + S: *n* = 5; LPS + NS: *n* = 4; LPS + S: *n* = 5).

We performed a PET scan to obtain potential functional correlates of the structural and metabolite data. PUS alone caused basal hypermetabolism of the cortex and hippocampus at the dorsal level, while brainstem regions showed basal hypometabolism (Fig. 5A). When combining this factor with MIA, the hypermetabolism shifted to the cortex and the brainstem hypometabolism disappeared (Fig. 5B), suggesting that prenatal LPS exposure modulates the effects of PUS on brain metabolism. Furthermore, cortical hypermetabolism induced by such a combination was notably stronger than the group that was not exposed to any hit (Fig. 5C).

No noticeable effects of MIA were observed on brain metabolic activity neither in the absence (Fig. S9A) nor in the presence of PUS (Fig. S9B).

### RNAseq analysis

Given their involvement in addiction-related phenomena, we selected the NAcc and dorsolateral striatum to perform our transcriptomic analyses.

### Nucleus accumbens

**Fig. 6:**
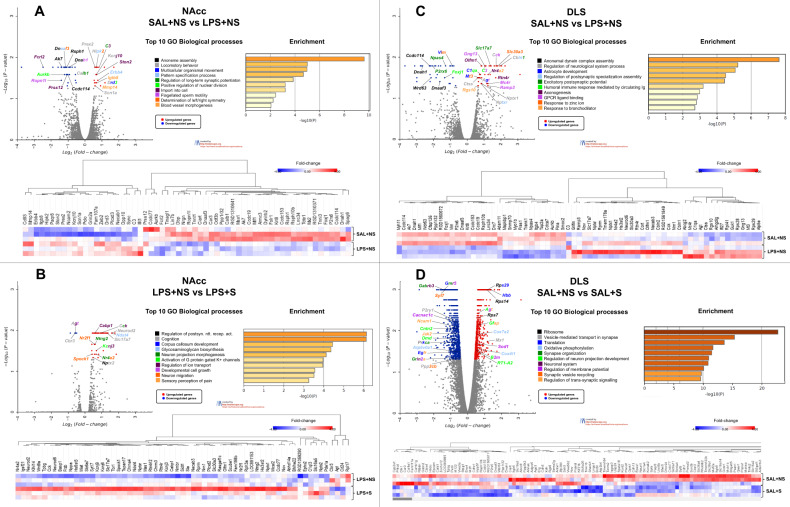
RNAseq analysis in the NAcc and dorsolateral striatum. The figure shows volcano-plot distributions of up- and down-regulated genes according to their *p* value and fold-change, the top 10 GO biological processes in which they are involved, associated bar charts of enriched terms coloured by *p* value (where the terms containing more genes show more significant p-value) and heatmaps showing fold-change values of differentially expressed genes in the NAcc (**A**, **B**) and dorsolateral striatum (**C**, **D**). In **D**, the heatmap corresponds to the dark grey area of the bar that represents the whole set of DE genes (SAL + NS: *n* = 3; LPS + NS: *n* = 3; SAL + S = 3; LPS + S = 3).

The analysis of the gene ontologies affected showed that MIA reduced the expression of different genes that clustered in separate groups of ontologies. On the one hand, it affected genes involved in the development of the nervous system, such as those regulating axoneme assembly, pattern specification or left/right symmetry. On the other, it affected several gene ontologies related to behavioural regulation and central nervous system function, such as ‘locomotory behaviour’, ‘movement’ and ‘long-term synaptic potentiation’ (Fig. 6A). As for the effects of MIA among stressed animals, the affected ontologies included synaptic functionality, neurodevelopment, and cognition (Fig. S10).

Interestingly, while PUS had no effects on its own, among LPS-exposed animals, PUS altered the expression of genes involved in the regulation of postsynaptic receptors activity, neurodevelopmental processes such as cell growth, neuron migration or neuron morphogenesis, and even in more complex processes such as ‘cognition’ and ‘sensory perception of pain’ (Fig. 6B). Tables S3–S5 provide more detailed information on the genes that belong to each ontology. Figure S12 shows the number of differentially expressed genes (DEGs) per condition and DEG overlap between experimental groups in the NAcc.

### Dorsolateral striatum

The gene ontology analysis showed that MIA induced and repressed the expression of several genes clustered in networks that, unlike the NAcc, had no apparent separation. In general, the top five regulated ontologies were involved in the development of the nervous system, such as ‘axoneme assembly’, ‘astrocyte development’, ‘axonogenesis’ or ‘neuron migration’. It also altered the expression of genes involved in biological processes relevant to the functionality of the nervous system, such as the regulation of postsynaptic specialisation assembly or the generation of excitatory postsynaptic potentials (Fig. 6C). These MIA-induced gene expression alterations were no longer observed among stressed animals. On the other hand, PUS alone altered the expression of nearly 2000 genes involved in a wide variety of biological processes, mainly at the ribosomal and translational machinery level, synaptic organisation and function and behaviour, among others (Fig. 6D and Table S2). However, MIA completely modulated these PUS-induced effects, and among LPS-exposed animals, PUS only affected the expression of genes involved in axoneme assembly (Fig. S11). Tables S6–S8 provide more detailed information on the genes that belong to each ontology. Figure S13 shows the number of differentially expressed DEGs per condition and DEG overlap between experimental groups in the dorsolateral striatum.

## Discussion

In addition to its disabling consequences, schizophrenia is usually associated with higher rates of substance use disorders [17]. To shed some light on this issue, we have studied the presence of cocaine addiction-like behaviour and the underlying alterations in brain structure and function in a two-hit animal model relevant to schizophrenia. Figure 1B shows a summary of the main results obtained.

### Cocaine addiction-like behaviour and associated processes

In our categorical analysis, MIA tended to increase the likelihood of acquiring cocaine self-administration; however, it did not affect drug intake among those animals that did acquire the self-administration behaviour. This absence of effects on cocaine intake is consistent with our previous reports that showed MIA had no impact on cocaine self-administration, extinction, progressive ratio performance, dose-response curves or cue-induced reinstatement [9, 10]. However, PUS remarkably affected drug intake over the acquisition sessions, lowering the amount of cocaine earned by animals exposed to stress. Previous reports in the literature have already suggested that social defeat stress during adolescence might delay the discrimination between active and inactive lever presses [18]. Still, no studies have indicated that the actual amount of self-administered cocaine could be lower than in non-stressed animals. Quite the contrary, experiments with unpredictable stress during adolescence showed a slightly higher preference for cocaine in adult rats in a two-bottle choice test [19]. Moreover, it has been recently documented that exposure to an early-life stressor such as maternal separation increased cocaine seeking in adult male mice by altering glutamatergic activity in the medial prefrontal cortex [20], suggesting that the type of stressor and the age at which animals are exposed to them are critical variables that determine the effects of early-life stress on cocaine reward.

It is noteworthy that the decreased cocaine self-administration due to stress that we found here was, however, absent in LPS-exposed rats, which displayed a similar amount of cocaine intake regardless of their stress experience. These counteracting effects of MIA are consistent with the contemporary notion that a prior hit may confer resilience or render subjects insensitive to the actions of a second hit later in life. For example, some of the effects of social isolation (the second hit in the following studies) are reduced by prior maternal separation [21], a high-fat diet [22], an immune challenge in adolescence [23], or, more recently, MIA [24].

After analysing acquisition, we examined other aspects of cocaine addiction-like behaviour, such as motivation for the drug (in a PR schedule) or compulsive seeking [13]. MIA increased the motivation for cocaine in the first session of the PR schedule, which is arguably not affected by the actual learning of the contingencies of the task and adequately reflects shifts in the motivational status of the animal. We then returned animals to FR1 and again observed the same pattern as in the acquisition phase, whereby PUS decreased cocaine intake. Interestingly, MIA reversed this effect, suggesting that these interactions, being long-lasting, only emerge under low-effort conditions. We then tested compulsive seeking, which was not affected by any of our experimental factors. Extended access conditions in self-administration paradigms induce a loss of control over cocaine intake and the emergence of several addiction-related phenomena [25]. Under these conditions (6-h FR1 sessions) we again observed the effects of PUS (a decrease in cocaine intake) and the modulatory effects of MIA on this phenomenon. Interestingly, this pattern of results did not extrapolate to the incubation of seeking. Nevertheless, our reported effects do not seem to be due to alterations in Pavlovian or instrumental learning per se, since we examined the performance of the animals on tasks involving these cognitive functions and found no changes due to MIA, PUS, or their interaction.

The specific pattern of addiction vulnerability observed in MIA animals (increased likelihood of acquiring cocaine self-administration and increased motivation) was not parallelled with changes in impulsivity, which was somewhat expected because impulsivity seems to predict the emergence of a complete addiction phenotype characterised not only by increased motivation but also by compulsive seeking and increased seeking when the drug is not available [26] (which in our design is captured by lever presses during signalled time-out periods, a variable that was not affected by MIA, PUS or their interaction).

### Neuroimaging studies

After characterising the addiction-like behaviour of the animals in this two-hit model, we turned to imaging techniques to gain further insight into the associated brain alterations. Given the relevance of this two-hit model to schizophrenia, we provide a supplementary discussion of the implications of our findings in the context of this condition (see Supplementary Information).

PUS decreased the volume of the right hippocampus. Interestingly, PUS also increased the metabolic activity in the right dorsal subicular compartment of the hippocampal formation, an effect that was prevented in MIA rats. Considering that the dorsal subiculum is necessary for cocaine-seeking and cocaine-taking [27], the abnormal activity observed in this structure could explain to some extent the persistent decrease in self-administration behaviour detected across most phases of the addiction-like behaviour examination that we have performed and the normalisation of this behaviour in rats with MIA experience. An additional explanation may rely on the observed increased dorsal striatal volume due to PUS evident only in rats with a history of MIA (especially in the left hemisphere). This effect was concomitant to a decrease in NAA + NAAG levels in MIA + PUS rats. NAAG is an agonist of the mGluR3 receptor and negatively modulates glutamate levels [28] therefore, the reduced levels of this peptide may result in increased glutamate levels, which is what we observed in a previous study in MIA + PUS rats where we used ex vivo 11 T MRS (which allowed us to have a separate measure of glutamate from glutamine peak) [29]. Considering all these pieces of evidence, it could be suggested that MIA + PUS rats show an acceleration of habit formation after the initial contact with cocaine that may render their responding for cocaine divorced from the (decreased) rewarding actions of the drug (as observed in PUS rats). This assertion is based on the striatal alterations that we observed (increased glutamate levels due to decreased inhibition of glutamate release parallelled with increased striatal volume), which would also be consistent with a recent report that suggests that glutamate dynamics in the left putamen of people with a diagnosis of cocaine use disorder are predictive of their reported habit-like behaviour (i.e., the automaticity dimension of the Creature of Habit Scale) [30].

Cortical choline compounds were reduced in PUS alone animals and an effect that was absent in MIA + PUS rats. The GPC + PCh peak has been traditionally interpreted as reflecting membrane turnover or myelin dynamics [31]. In the context of cocaine addiction, it has been shown that cocaine users have higher cortical choline levels; however, in that study, a potential confounding effect of recent cocaine use cannot be ruled out [32]. Our data may suggest that these metabolites could be driving cocaine use rather than being a consequence. Indeed, PUS decreased the levels of this peak in our MRS study and decreased cocaine self-administration, while MIA experience prevented both effects of PUS. This possibility opens an exciting avenue for further research that should be explored in the future.

Some of the brain alterations observed are also relevant to schizophrenia and, as such, are discussed in the Supplementary Discussion.

### Transcriptomic studies

Rats with LPS exposure during prenatal development tended to acquire cocaine self-administration faster. Moreover, these rats also showed increased motivation for cocaine. We have observed that MIA affected a gene ontology with potential implications for this pattern of results in the NAcc: ‘regulation of long-term synaptic potentiation’, which may affect the capacity for plasticity of the NAcc upon exposure to cocaine. For example, the gene for Neurogranin (*Nrgn*), present in this ontology (Table S3), responds to cocaine in several ways. For example, it is induced by cocaine in neural progenitor cells [33] and it is related to anhedonia in cocaine users [34]. Moreover, neurogranin in the NAcc also regulates the sensitivity to other drugs, such as alcohol [35]. Another gene in the ontology, *Calb1*, coding for the protein calbindin, is also responsive to cocaine self-administration in the NAcc [36]. Accordingly, the expression of these cocaine-responsive genes may be suggested to render these individuals more vulnerable to acquiring cocaine self-administration.

On the other hand, PUS did not affect gene expression in the NAcc, which contrasts with its potent effects on cocaine self-administration in those animals that acquired the behaviour. We argue that other brain regions more susceptible to stress, such as the bed nucleus of stria terminalis [37] or the central amygdala [38, 39], may directly or indirectly influence cocaine reward. However, the abolishment of the PUS-induced reduction in cocaine intake observed in rats exposed to MIA and PUS may be related to transcriptomic changes in the NAcc induced by the combination of both hits. Indeed, as opposed to the absence of effects in controls, PUS modulated several gene ontologies in MIA animals that could account for the restoration of cocaine intake. These categories include ‘regulation of postsynaptic neurotransmitter receptor activity’, ‘regulation of ion transport‘ (including ‘activation of G protein gated potassium channels’) and ‘cognition’. Some members of the pentraxin gene family that we found to be modulated here (*Nptxr*|*Nptx1*|*Nptx2*) (Table S5) are involved in anxiety and responses to stress [40], which support their modulation in stressed animals. More importantly, they are involved in synaptic processes related to cocaine self-administration, cocaine-induced rearrangements of AMPA receptor trafficking and also in the modulation of cocaine-associated memories [41–43]. According to these data, this family of proteins could be partially responsible for the increased self-administration observed in LPS-exposed stressed rats compared to their controls. Other ontologies affected, such as that involving potassium channels, could also be relevant in the explanation of the restoration of cocaine intake in LPS-exposed stressed animals. Indeed, this family of potassium channels are also involved in cocaine responses [44–46].

PUS profoundly affected the dorsal striatal transcriptome. 1938 DEGs were found in this area in animals that underwent PUS. However, this transcriptomic ‘scar’ did not form in rats with MIA. The analysis of the gene ontologies affected by PUS points to profound modifications in the translational machinery of striatal cells. Indeed, the ‘Ribosome’ ontology showed a remarkable impact in PUS-exposed rats, together with the ‘Translation’ category and other plasticity-related ontologies such as ‘Synapse organisation’. It seems that PUS affects striatal function by perturbing protein translation of synaptic-related proteins.

Given that in addition to the NAcc, the dorsal striatum also regulates cocaine intake under fixed-ratio 1 conditions [47], it is tempting to speculate that this structure could be aberrantly imposing a break or decreasing its goal-directed influence (see discussion of neuroimaging findings above) on cocaine-taking responses. In support of this notion, several addiction-associated genes were down-regulated by PUS in the dorsolateral striatum, which could account for PUS’s effects on cocaine self-administration at this early stage. Some examples of these genes include *Oprk1* (Kappa opioid receptor) [48], *Gabra2*, (GABA_A_ Receptor Subunit Alpha2) [49], *Grin2A* (Glutamate Ionotropic Receptor NMDA Type Subunit 2A) [50], *Chrnb2* (Cholinergic Receptor Nicotinic Beta 2 Subunit) [51] or *Gabra4* (Gamma-Aminobutyric Acid Type A Receptor Subunit Alpha4) [52]. Interestingly, these gene alterations were obliterated in stressed animals with a MIA background, which could also explain the normalisation of cocaine intake when both hits are combined.

We also found interesting patterns of gene expression data as a consequence of PUS and its interaction with MIA in the NAcc or dorsolateral striatum that are relevant to schizophrenia and discussed in the Supplementary Discussion.

### Caveats and concluding remarks

There are some caveats to our study that we would like to acknowledge. First, we only focused on the male population, mainly because our previous data suggested that MIA only had effects on the male sex and also because of the complexity of our design. Second, we have a small sample size in our PET and sequencing data, so these results should be replicated in future studies. Third, we did not obtain RNAseq data from the hippocampus or dorsal subiculum, where the imaging data pointed to exciting interactions, so future sequencing experiments should focus on these regions. Fourth, we only found a trend towards significance in the PPI data, more specifically in the 12 dB 30 ms condition (Fig. S1 and Table S1). Following the work of Giovanoli and coworkers, it is essential to mention that in any two-hit study, the first hit should induce a subthreshold effect so that the synergistic effect of the second hit may be observed. Moreover, prepulse inhibition of the acoustic startle response is just one of the many behavioural dimensions affected by maternal immune activation procedures. It is not the only factor that is reflective of alterations present in schizophrenia. Indeed, social interaction deficits, working memory impairments, and enhanced locomotor response to an amphetamine challenge are behavioural dimensions typically affected by gestational immune activation [53, 54]. In addition, other researchers have reported an absence of PPI deficits in MIA experiments in the presence of other behavioural disturbances indicative of the presence of the phenotype [55–57]. Lastly, the PPI response is very variable and its alteration by gestational LPS exposure is likely to be influenced by several factors such as the immune response generated, the batch of LPS used, etc.

In conclusion, the results of the present work suggest that the interactions between prenatal immune activation and stress around puberty are complex and bidirectional. Concerning the critical question of cocaine addiction in individuals suffering from neurodevelopmental disorders such as schizophrenia, we argue that stress around puberty, instead of unmasking the latent vulnerability to addiction, may induce an anhedonic status that would be prevented by MIA experience. While the neural networks responsible for these interactions likely involve diversely distributed brain nodes, the neuroimaging and sequencing techniques employed here suggest an important role for the hippocampal formation and the dorsal striatum.

## Supplementary information

Supplemental Material

Supplemental Table 3

Supplemental Table 4

Supplemental Table 5

Supplemental Table 6

Supplemental Table 7

Supplemental Table 8
