# Supplementary material for: Interaction between maternal immune activation and peripubertal stress in rats: impact on cocaine addiction-like behaviour, morphofunctional brain parameters and striatal transcriptome
Source: Transl Psychiatry. 2023 Mar 8;13:84. doi: 10.1038/s41398-023-02378-6 (PMC9995324; doi:10.1038/s41398-023-02378-6)
Supplement: Supplementary file 1 — Supplemental Material [file 41398_2023_2378_MOESM1_ESM.docx]

**Supplemental Information**

**Interaction between maternal immune activation and peripubertal stress in rats: impact on cocaine addiction-like behaviour, morphofunctional brain parameters and striatal transcriptome**

## Index

Supplementary Materials and Methods

Supplementary Results

Supplementary Discussion

Supplementary Figures S1-S13

Supplementary Tables S1-S2

# Supplementary Materials and Methods

*Experimental animals*

Experiments were performed on the male offspring of 14-week-old male and 12-week-old female Sprague-Dawley rats obtained from Charles River (France). Animals were kept in a temperature and humidity-controlled environment (23 °C/50–60%), artificial light (12 h/12 h light/dark cycle, lights on at 8 p.m.), ad libitum access to food (commercial diet for rodents A04: Panlab, Barcelona, Spain) and tap water, unless otherwise specified. Rats were housed in transparent Plexiglas cages (48.3 cm length x 26.7 cm width x 20.3 cm height). All the procedures performed were compliant with European Union guidelines for the care of laboratory animals (EU Directive 2010/63/EU governing animal experimentation) and approved by the Ethics Committee of UNED and the Autonomous Community of Madrid (PROEX 078/18).

*Maternal immune activation*

Male and female rats were mated one week after arrival at the animal facility. Vaginal smears were taken daily from the breeder females, and pregnancy was determined by the presence of sperm in the vaginal smear (day 0 of pregnancy). LPS (from Escherichia coli 0111: B4 [Sigma-Aldrich]) dissolved in 0.9% NaCl was intraperitoneally injected to pregnant rats at a dose of 100 mg/kg/ml on gestational days (GD) 15 and 16. This dose was chosen based on previous studies showing that it does not significantly alter the percentage of dam survival and that it only has marginal effects on PPI^1–3^. Moreover, in a previous study from our group, we showed that this dose had no consequences on social interaction or cocaine self-administration^4^. In considering all these pieces of evidence it seems clear that this dose is suitable for a two-hit study where a single insult should not have strong effects on its own. The control group consisted of pregnant rats submitted to the same treatment schedule with saline injection instead of LPS. Whenever possible, litter size was standardised to 8-12 pups, culling the animal surplus. We marked the pups according to their prenatal treatment with a tattoo on the paw and ensured that each dam had an equal number of LPS and saline-exposed pups. In doing so, we homogenized any potential effect of prenatal treatment on maternal behaviour that could affect the development of the offspring.

*Peripubertal unpredictable stress*

Litters were left undisturbed until postnatal day (PND) 21 when they were weaned and grouped in sets of 2-3. Each set belonged to the same litter and treatment. Between PNDs 28 and 38, we exposed the male offspring to peripubertal unpredictable stress (PUS). This stage of development is a critical period known to be highly sensitive to the disrupting effects of traumatizing events relevant to neuropsychiatric disorders^5^. Given that in our previous work we only found MIA-related effects in the male offspring^4^, in this work we decided to test the males only. The stress protocol included five distinct stressors, that were sequentially applied every other day, in a randomized order: 1) Stress by agitation (30 min in an orbital shaker at 100 rpm). 2) Stress by immobilization (45 min in a cylindrical restrainer under bright light). 3) Water deprivation for 16 h. 4) 10 min of a forced swimming session in a water tank of 40 cm high x 18 cm in diameter at a temperature of 22 ± 1 ºC and a depth of 30 cm. 5) Constant changes of the home cage (five cage changes, with new sawdust, during the dark cycle at random intervals). Non-stressed controls received handling by the same researcher and on the same days as the stressed subjects. This protocol was adapted from the work of Giovanoli et al., ^5^ who showed that PUS unmasked several psychological and neurobiological consequences of prenatal immune activation such as latent inhibition and PPI deficits or dopaminergic hyperactivity and increased immune reactivity in the hippocampus.

*Experimental Design*

Four different experimental groups resulted from the manipulations described above: saline exposed animals (SAL+NS), saline & PUS exposed animals (SAL+S), LPS exposed animals (LPS+NS) and LPS & PUS exposed animals (LPS+S). All the studies that will be described hereafter were carried out in independent batches of animals, except for the PPI test, which was performed in all of them. The exact number of litters per experiment is as follows: Self-administration experiment: Saline: N=9; LPS; N=9; Pavlovian and instrumental conditioning experiment: Saline: N=9; LPS; N=12; 2-CSRTT experiment: Saline: N=4; LPS; N=3; MRI experiments: Saline: N=5; LPS; N=5; PET experiment: Saline: N=4; LPS; N=4; RNAseq experiment: Saline: N=4; LPS; N=4. Figure 1A shows an outline of the experimental design employed. The final sample size of each of the four experimental groups is indicated in the figure footnotes and Table S1.

*Experimental Procedures*

Prepulse inhibition of the acoustic startle response (PPI)

At PND70-73, PPI of the acoustic startle was measured in a non-restrictive Plexiglas cage (28x15x17 cm) containing a vibration-sensitive platform (Cibertec), enclosed in a sound-attenuating chamber, and a set of two speakers located above the cage. Rats were habituated for 7 min with a background noise of 65 decibels (dB), which continued throughout the session. Animals were exposed to 6 pulse-alone trials at the beginning and the end of the session, to stabilize the startle response and to calculate the habituation percentage (these pulses were not included in the PPI calculations). The session was composed of 35 different trials: ten 120 dB pulse-alone trials, five null trials with no stimulus and twenty pulses preceded by a prepulse of 69- or 77-dB intensity (4 or 12 dB above the background noise, respectively) with an interval of 30 or 120 ms. The duration of the test was 20 min approximately. Prepulse inhibition is expressed as the % PPI and calculated using the following formula: 1 − [startle amplitude on prepulse + pulse trial/mean startle amplitude on pulse-alone trials]) x 100. The percentage of habituation is expressed as 100 x [(Mean of first pulse-alone block − Mean of last pulse-alone block) ⁄ Mean of first 6-pulse alone block].

Pavlovian and Instrumental conditioning programs

At PND90, food was removed, and body weight was controlled so that it remained between 90-95%. Food was provided after each experimental session. The Pavlovian learning protocol consisted of eight daily sessions. Each session consisted of 4 cycles of 12 minutes. In each cycle, animals were exposed to 4 minutes of a continuous “tone” stimulus, 4 minutes of an intermittent “click” stimulus and 4 minutes in the absence of stimuli between both. During exposure to one of the auditory stimuli (either tone or click), pellets were dropped according to a 5-second variable time program (different time intervals with an average time of five seconds). The conditioned stimulus "CS+", signalled cue-delivery periods whereas the absence of pellets was signalled by the "CS- " stimulus; in both cases head entries (HEs) to the feeder were recorded. The 4-minute interval between CS + and CS- was the ISI (inter-stimulus interval), where only the white noise produced by a fan designed to isolate the sound boxes from the outside was maintained. CS+ and CS- were counterbalanced between subjects to avoid attentional biases derived from the unconditioned excitatory capacity of each stimulus, as well as the order in which they were presented. During this protocol, Skinner boxes had no visible operating levers.

The instrumental learning protocol was performed after the Pavlovian training and consisted of seven daily sessions. Animals had two levers available. Pressing on one of them produced pellet release so that this lever was called "active" while pressing the other one (“inactive”) had no consequence. Once the active lever was pressed, a 5- second timeout began with no pellets. Sessions ended after 30 minutes or after 30 pellets had been earned. Animals completed one fixed ratio 1 (FR1) session, three variable ratio-5 (VR5) sessions and three variable ratio-10 (VR10) sessions. No light cues were presented during the sessions. White noise was present during the sessions to dim the noise from outside the operant chambers.

Two-choice serial reaction time task (2-CSRTT)

Once the PND90 was reached, food was removed, and body weight was controlled so that it remained between 90-95% of free-feeding values. Food was provided after each experimental session. The 2-CSRTT employed here was an adaptation from the 5-choice serial reaction time task protocol^6^. We used Skinner boxes (Med Associates) equipped with a feeder with entry detectors, two retractable levers placed at both sides of the feeder, and two small lamps to present cue lights above the levers. Before the beginning of the protocol, a brief training was implemented to associate the light stimulus placed on top of each lever with the release of one pellet when the lever was pressed. This training consisted of two sessions, one for each lever, with a 30-minute or 30 pellets limit. The actual 2-CSRTT protocol consisted of 13 different stages, and each one was composed of 100 serial trials. Light stimulus duration was progressively reduced throughout stages from 30 to 0.5 seconds. Response time (or time to respond since light stimulus appeared) was also diminished from 30 to 5 seconds, while inter-trial interval (ITI) (or time elapsed between trials) increased from 2 to 9 seconds. Trials started when the animal introduced its head inside the feeder, and the signalled lever was alternated randomly during these trials. Correct answers were rewarded with a food pellet, while incorrect answers, omissions (absence of response) and premature responses (lever presses before light stimulus presentation) were punished with a 5-second time-out, where the Skinner box remained in darkness, and no rewards were available. Each session ended after 100 trials were completed or after 30 minutes, whichever occurred first, and accuracy and omissions percentages were calculated to determine if the animal could progress to the next stage^6^. Once stage 12 was reached, animals remained in this phase until six consecutive sessions were successfully completed, to stabilize performance. The criterion applied in this phase was

≥75% accuracy and ≤20% omissions. Afterwards, three long ITI sessions (inter-trial interval was increased to 9 seconds) were performed, separated by two baseline sessions each. This was done to elicit impulsive behaviour^7^. Between 25 and 45 sessions were carried out until all the animals reached this point.

Neuroimaging studies

Magnetic resonance imaging (MRI) and diffusion tensor imaging (DTI)

At PND90, MRI and DTI studies were executed at the Biomedical Research Institute "Alberto Sols" (CSIC-UAM, Madrid, Spain). Experiments were performed on a Bruker PharmaScan system (Bruker Medical Gmbh, Ettlingen, Germany) using a 7.0 Tesla horizontal-bore superconducting magnet, equipped with a 1H selective quadrature 40 mm coil and a 90 mm-diameter gradient insert (36 G/cm maximum intensity). All data were acquired using a Hewlett-Packard console running Paravision 5.1 software (Bruker Medical Gmbh) operating on a Linux platform. Rats were placed into the centre of the radiofrequency volume coil and positioned in the magnet under continuous anaesthesia inhalation via a nose cone. A respiratory sensor connected to a monitoring system (SA Instruments, Stony Brook, NY) was placed under the abdomen to monitor respiration rate and depth. Animals were anaesthetized with a 2% isoflurane-oxygen mixture in an induction chamber and the flow of anaesthetic gas was constantly regulated to maintain a breathing rate of 50 +/- 20 beats per minute. T2-weighted (T2-W) spin-echo anatomical images were acquired with a rapid acquisition with relaxation enhancement (RARE) sequence in axial and coronal orientations and using the following parameters: TR: 3000 ms, TE: 44 ms, RARE: factor 8, Averages: 3, FOV: 3,5 cm, Acquisition matrix: 256 × 256 corresponding to an in-plane resolution of 136 × 136 μm2, Slice thickness: 1,5 mm, Number of slices: 18 for axial and 8 for coronal images. Diffusion-weighted images were acquired with a spin-echo single-shot echo-planar imaging (EPI) pulse sequence using the following parameters: TR: 3500 ms, TE: 40 ms, Averages: 1, Diffusion gradient duration: 3,5 ms, Diffusion gradient separation: 20 ms, Gradient directions: 7, Acquisition matrix: 96x96 and zero-filled in k-space to construct a 128 × 128 corresponding to an in-plane resolution of 273x273 μm2, B values: 100 s/mm2 and 1400 s/mm2, Slices thickness 1,5 mm. Fractional anisotropy, mean diffusivity, trace, eigenvalues and eigenvector maps were calculated with a homemade software application written in Matlab (R2007a). Values were extracted from maps using regions of interest (ROIs) with Image J software. The volumetric quantification of the different brain structures or ventricles was normalized to the brain volume of the MRI slice containing the analysed region, ruling out potentially confounding effects of the differences in brain volume observed as a consequence of MIA.

*In vivo* proton magnetic resonance spectroscopy (1H-MRS)

Immediately after conducting the MRI and DTI analysis, an *in vivo* 1H MRS study was also performed in the Biomedical Research Institute "Alberto Sols" (CSIC-UAM, Madrid, Spain). Two brain regions were selected for this study: cortex and striatum. A Point- REsolved Spatially Spectroscopy (PRESS) was used, combined with a variable power radiofrequency (VAPOR) water suppression and employing the following parameters: TR: 3000 ms, TE: 35 ms, Averages: 128, Voxel volume: 3 mm3. First and second-order shims were automatically adjusted with a fast, automatic shimming technique by mapping along projections (FASTMAP) in a large voxel (4 mm3). The spectra were automatically analysed using LCModel software^8^, 6.2-OR version (Oakville, ON; Canada). Only the peak concentrations obtained with a standard deviation lower than 20% were accepted.

Positron emission tomography/computed tomography (PET-CT)

Once PND90 was reached, PET-CT studies were performed at the Radioisotopes for Biomedicine research group of the Center for Energy, Environmental and Technological Research (CIEMAT) in Madrid, Spain, using a small-animal PET-CT scanner (Argus PET/CT, SEDECAL, Madrid, Spain). PET (400–700 KeV energy window, 45 min static acquisition time) and CT studies (45 kV voltage, 150 μA current intensity,

8 shots, 360 projections, standard resolution) were performed 30 minutes after inoculation of 15.4±1.4 MBq of [18F]-2-fluoro-2-deoxy-d-glucose (18F-FDG) via the tail vein. Animals were anaesthetized by inhalation of 2–2.5% isoflurane in 100% oxygen. PET-CT image reconstruction was accomplished using a 2-dimension ordered subset expectation maximization (2D-OSEM) algorithm (16 subsets and 3 iterations), with random and scatter correction. The relatively poor spatial resolution of PET-CT imaging and the difficulty in identifying anatomical regions was minimized by co-registering PET- CT images to same-subject MR images^9^. Brain masks (corresponding to hippocampus, caudate bodies, prefrontal cortex, cortex and whole-body) were manually segmented on the MR template and applied to their corresponding PET-CT study. Voxel value normalization consisted of standardizing PET intensity data to a brain region without statistically significant differences between groups obtained by an iterative method (see^10^ for further details). We used a full ANOVA design followed by pair-wise comparisons after significant main effects. Statistical comparisons (p<0.01 uncorrected) were performed with Statistical Parametric Mapping (SPM) software [(http://www.fil.ion.ucl.ac.uk/spm/software/spm12/).](http://www.fil.ion.ucl.ac.uk/spm/software/spm12/)) Registered PET images were smoothed with a gaussian kernel of 2.5 times the voxel size of full width at a half maximum (FWHM) and masked in order to exclude extracerebral voxels from the analyses. Only clusters larger than 50 adjacent voxels were considered to minimize the effect of type I errors.

RNAseq

Once PND90 was reached, animals were decapitated for the dissection of different brain structures, under isoflurane anaesthesia. All the dissection material was autoclaved and treated with RNase*Zap*™ (Invitrogen) to avoid RNA degradation by RNases. In addition, water and saline solutions containing diethyl pyrocarbonate (Sigma- Aldrich) (1:500) were used to wash the dissecting material and tissue, respectively. An acrylic brain matrix for a 300 g - 600 g rat and razor blades were used for brain slicing, using the Paxinos atlas^11^ as a reference. The entire procedure was carried out at 4º C to prevent tissue degradation. The dissected samples were preserved in RNAlater™ Stabilization Solution (Invitrogen) for one day at -20°C and subsequently stored at -80°C. RNA extraction was performed using the RNeasy Mini Kit (Qiagen). RNA-Seq analysis was carried out in the Genomics Unit of the Madrid Science Park. RNA integrity number (RIN) and concentration were evaluated by employing an Agilent 2100 Bioanalyzer using an RNA 6000 nano LabChip kit. Libraries were prepared according to the “NEBNext Ultra Directional RNA Library Prep kit for Illumina” (New England Biolabs) instructions. Chapter 1: Protocol for use with NEBNext Poly(A) mRNA Magnetic Isolation Module” indications were followed. Before starting the protocol, the total RNA input yield was 1

µg. A 14-cycle PCR was used to obtain the library amplification included in the mentioned protocol. Libraries were validated and quantified by an Agilent 2100 Bioanalyzer using a DNA7500 LabChip kit. An equimolecular pool of libraries was titrated by quantitative PCR using the “Kapa-SYBR FAST qPCR kit forLightCycler480” (Kapa BioSystems) and a reference standard was used for quantification. The pool of libraries was denatured before being seeded on a flow cell at a 2,2 pM density, where bunches were formed and sequenced using a “NextSeq™ 500 High Output Kit”, in a 1x75 single read sequencing run on a NextSeq500 sequencer. Once the sequencing process was finished, the Illumina Analysis Space tool was used to map and locate the different sequences in the reference genome, generating alignment files in “. bam" format. These files were used to perform a differential expression analysis using the CUFFDIFF tool, which counted the RNA expression of each gene, normalized by its size and by the global RNA expression of each sample, and made a comparison between groups applying a False Discovery Rate (FDR) correction (q<0.05). We then used the Metascape (https://metascape.org/gp/index.html#/main/step1) resource to analyse the enrichment in specific gene ontologies for each comparison.

All RNAseq data sets generated and/or analysed during the current study were added to the Gene Expression Omnibus (GEO) under the accession number GSE185195.

# Supplementary Results

Prepulse inhibition of the acoustic startle response

We only found a trend in LPS-exposed animals to show impaired PPI in the 12 dB 30 ms condition (p=0.056) suggesting a latent deficit, but, contrary to our expectations, no interaction was observed between MIA and PUS (see Table S1). Other than this, no significant global effects of MIA, PUS or their interaction were observed in any of the experimental conditions used in the PPI test (Figure S1).

## Analysis of Pavlovian and instrumental conditioning

To rule out potential alterations in Pavlovian or instrumental conditioning that may have affected the self-administration data, we analysed if prenatal immune activation or PUS these two forms of learning. Animals progressively learnt both tasks, as revealed by the significant effects of the Pavlovian (F1,56=20.181; p=0.000; η2p=0.265) (Figure S3, A) and Instrumental (F1,56=39.638; p=0.000; η2p=0.414) (Figure S3, B) sessions factors, however, no significant differences were due to prenatal immune activation, PUS or their interaction.

## Motor impulsivity

Given that impulsivity is an endophenotype that confers vulnerability to addiction, we asked if prenatal immune activation, PUS or their interaction affected impulsivity. Impulsive behaviour, as captured by the 2-CSRTT, was not modified by prenatal immune activation or PUS. Indeed, no significant effects were observed by prenatal immune activation, PUS or their interaction in % premature responses in any of the sessions (Figure S4, A), nor the normalized responses during long-ITI sessions (Figure S4, B and C). A significant effect of the Sessions factor was found in the correct responses (F1,27=20.177; p=0.000; η2p=0.428), incorrect responses (F1,27=4.756; p=0.001; η2p=0.150), omissions (F1,27=3.331; p=0.021; η2p=0.118), perseverative responses (F1,27=3.305; p=0.010; η2p=0.109) and premature responses (F1,27=33.707; p=0.000; η2p=0.555), however, no significant effects of the prenatal immune activation or PUS factors or their interaction were detected, ruling out potential deficits in sustained attention in these animals (Figure S4, C-G). In addition, no significant differences were observed in the number of sessions to reach stage 12 (Figure S4, H).

## MRI-assisted volumetry

## No significant effects of prenatal immune activation, PUS or their interaction were found in cerebellar, amygdalar and NAcc volumes (Figure S5, A-C), nor in the fourth ventricle, third ventricle, lateral ventricles, cerebral aqueduct, and total ventricular volumes (Figure S6, A-F).

## PET

## No noticeable effects of prenatal immune activation were observed on brain metabolic activity neither in the absence (Figure S9, A) nor the presence of PUS (Figure S9, B).

## RNAseq data

Nucleus Accumbens

Illumina's RNA sequencing reports showed that MIA induced the differential expression of 60 genes in non-stressed animals and this amount was reduced to 53 among stressed rats. Surprisingly, only three genes were shared between the two comparisons (*Aurkb*, *Car3* and *Tnnt1*, downregulated in all cases), suggesting that PUS changes the gene expression programmes induced by MIA in the NAcc. PUS did not affect gene expression in control rats but was associated with the differential expression of 63 genes among animals subjected to MIA (Figure S12). There were 36 differential expressed genes (DEGs) that responded to one hit (MIA or PUS) on specific levels of the other hit: 35 shared DEGs between the SAL+S vs LPS+S comparison and the LPS+NS vs LPS+S comparison, and 1 DEG shared between the SAL+NS vs LPS+NS comparison and the LPS+NS and LPS+S comparison (Figure S12). MIA increased the expression of the *Sync* gene (coding for Syncoilin) (Table S2) and reduced the expression of several genes (*Car3* -carbon anhydrase-, *Fcrl2* - Fc Receptor Like 2- and *Folr1* - Folate Receptor Alpha- being with the ones with highest fold-change).

Dorsolateral Striatum

In the dorsolateral striatum, MIA induced the differential expression of 69 genes while this effect was completely obliterated among animals subjected to PUS. Surprisingly PUS alone induced the expression of 1938 DEGs, but this effect was almost completely abolished in rats with a history of MIA (only 15 DEGs were found in the LPS+NS vs LPS+S comparison) (Figure S13). Only five DEGs associated with PUS were shared among MIA-exposed and non-exposed rats (*Aurkb*, *Ccded153*, *Dnah1*, *P2rx6*, and *Rsph10b*). Noteworthy, these five genes were down-regulated in PUS rats but changed the direction of their expression (to up-regulation) when PUS occurred in MIA-exposed rats, suggesting profound epigenetic modifications. There were 37 shared DEGs among those induced by MIA (SAL+NS vs LPS+NS comparison) and PUS (SAL+NS vs SAL+S comparison). Lastly, 14 DEGs were found both due to MIA in non-stressed rats and due to PUS in MIA-exposed animals, but in opposite directions (Figure S13). In general, it seems that the dorsolateral striatum is more vulnerable to the effects of PUS than the NAcc and that it is more sensitive to the bidirectional interactions between MIA and PUS.

# Supplementary Discussion

### Brain imaging alterations in the context of schizophrenia

We will first discuss the effects of maternal immune activation (MIA) on its own and then examine MIA-PUS interactions. We found evidence for smaller whole brain volume in the rats exposed to LPS during gestation. This decrease in whole brain size (previously undocumented in LPS-induced MIA models) cannot be due to a concomitant decrease in body size because these animals had slightly increased body weight (mean±SD g: SAL+NS= 387.375±18.700; SAL+S=373.563±16.950.

LPS+NS=410.937±23.420; LPS+S= 421.500±30.833). Even if decreased whole brain volume is a general landmark in schizophrenia^12^, general reductions in whole brain volume are scarce in MIA experiments. Indeed, there are some MRI studies in the MIA literature that, relying on TLR3 activation via poly I:C (and hence mimicking viral infections rather than bacterial infections), have shown reductions in brain volume; however, in one of them these reductions were concomitant with increases in the volume of other areas of the brain^13^ and, in another study, the decrease in general brain volume was transient, emerging on PND35 and disappearing upon reaching adulthood^14^. Moreover, other poly I:C MIA studies have not found evidence for decreased whole brain volume^15–19^. Therefore, the results obtained with our specific parameters and immunogen provide further support for the notion that the reduction in whole-brain volume could indeed be a neurodevelopmental trait associated with schizophrenia, and not a consequence of the antipsychotic medication or other correlated variables, such as adverse life events or drug use. We also found evidence for increased MD in rats with gestational exposure to LPS. This increase in MD could point to reduced cellular and synaptic complexity^20^ and may imply a delay in the maturational processes that occur in the hippocampus. In addition, these preclinical findings also provide support to the evidence of an increase in MD in the hippocampus of people with a diagnosis of schizophrenia^21^, increasing the potential usefulness of this imaging landmark as an early diagnostic marker of the disease, even if the whole symptom clusters have not fully emerged. We did not detect any alterations due to MIA in brain metabolic activity in our PET experiments or the metabolite profile of the cortex or dorsal striatum in our 1H-MRS studies. These results contrast with previous literature and this divergence may be resulting from the different immunogens used in the studies. Indeed, while we used LPS in the present experiments, which mimics a bacterial infection, all previous reports have used poly I:C^17,22–27^. This is important in the context of understanding the differential effects that viral and bacterial infections may have on the developing brain.

While the effects of MIA on its own are interesting to examine brain alterations that may underly the susceptibility to schizophrenia and associated neurodevelopmental disorders, the addition of a second hit may be more relevant for the actual onset of the condition. In this context, we have observed very interesting MIA * PUS interactions. For example, MIA induced an increase in the striatal volume only in stressed animals which, however, was paralleled by a potential decrease in neuronal density (as indicated by decreased NAA+NAAG concentrations), suggesting that the higher volume of this structure in MIA-PUS rats may be more likely due to glial proliferation. Metanalysis of striatal NAA levels have not found consistent evidence for reduced levels of this metabolite in the basal ganglia in schizophrenia^28^, however, our findings with the animal model used here, together with our previous evidence for increased glutamate levels in the striatum of MIA+PUS rats^29^, suggest that further investigations are needed to elucidate the actual role of these striatal alterations in the context of schizophrenia-spectrum disorders.

### Gene expression changes relevant to schizophrenia

While MIA alone did not seem to induce relevant gene expression changes in the nucleus accumbens (NAcc) for schizophrenia (it affected genes related to the assembly of the axoneme and cilia-related genes), the effects of MIA in stressed animals shifted towards alterations of gene expression that involved a set of ontologies relevant for neurodevelopmental disorders. Indeed, categories important for the regulation of the synaptic structure, the transport of aminoacids such as glutamate, axonogenesis or cognition were significantly enriched. At the individual gene level in the NAcc, there were some genes with important associations to schizophrenia that were affected in MIA+PUS rats and that were not modulated in rats with MIA but not stress experience. Some examples of these genes are *Rtn4r* (Reticulon 4 Receptor) (which was also modulated by MIA in the dorsolateral striatum)^30–32^, *Tbr1* (T-Box Brain Transcription Factor 1) (modulated also by PUS but only among MIA-exposed rats)^33,34^, *Bdnf* (Brain-Derived Neurotrophic Factor)^35,36^ or *Slc17a7* (Solute Carrier Family 17 Member 7 or vesicular glutamate transporter 1)^37^. This transcriptomic landscape in the NAcc induced by the combination of MIA and PUS could therefore provide susceptibility to schizophrenia even if the symptoms of the disease have not fully emerged (for example, in the present study, the documented decrease in PPI was at the threshold of significance, suggesting that the full emergence of the disease had not completed).

The dorsal striatum is also a structure that is affected in schizophrenia^38,39^. Here, similarly to the NAcc, MIA also affected the axoneme assembly ontology, however, it also modulated other categories that are more relevant to neurodevelopmental disorders such as the regulation of neurological system processes (including important genes such as *Cck* which codes for the peptide cholecystokinin) or the regulation of the excitatory postsynaptic potential (which also includes relevant genes to schizophrenia such as *Slc17a7* which codes de vesicular glutamate transporter 1 protein or VgluT1). At the individual level, an important gene modulated by MIA was *Rtn4r* which, as stated above has been associated with schizophrenia and, in the NAcc, required the combination of MIA and PUS to be up-regulated. MIA also up-regulated *Cck* in the dorsolateral striatum, a peptide that has been involved in schizophrenia^40^. Indeed, adequate levels of cholecystokinin in the striatum have been suggested to control the interaction between cognition and reward circuitry, which seems to be crucial in schizophrenia^41^. The last gene that we would like to highlight regarding MIA effects in the striatum is *Nr4a2* which codes for the Nuclear Receptor Subfamily 4 Group A Member 2 protein (also known as *Nurr1*). This protein is a transcription factor implied in the differentiation, maturation, and survival of dopaminergic neurons and also has a role in regulating the expression of several proteins important for the synthesis and/or regulation of dopamine (DA)^42,43^ and was involved in the attentional deficits of patients with schizophrenia in one study^44^. As a whole, these results suggest that MIA in the striatum (as opposed to what is observed in the NAcc) can induce, on its own several gene expression alterations that may confer vulnerability to schizophrenia.

However, as stated in the previous section, the most notable effect in the striatum was the profound striatal alterations induced by PUS. 1938 DEGs were affected by stress in this structure. Some of the relevant ontologies, with potential implications for neurodevelopmental disorders, were those related to synapse organization, neuron projection development, trans-synaptic signalling or behaviour. Several of the genes in these categories belonged to the glutamatergic or GABAergic systems and have been previously shown to be involved in schizophrenia. Some examples are *Slc1a1, Grin2a*, *Gabra2, Gabrg2*, *Gria1*, *Grik2* or *Gad2.* Interestingly, when MIA and PUS were combined, the striatal transcriptomic landscape completely changed. PUS among animals exposed to MIA no longer affected the previously mentioned categories and biased the effects toward a specific set of genes related to the regulation of axoneme assembly. Moreover, at the induvial gene level, there were no genes with a strong involvement in neurodevelopmental disorders. Hence, at least at the striatal transcriptome level, it seems that MIA is protecting against the deleterious effects of stress with regards to the susceptibility to schizophrenia, two of the most severe neurodevelopmental disorders.


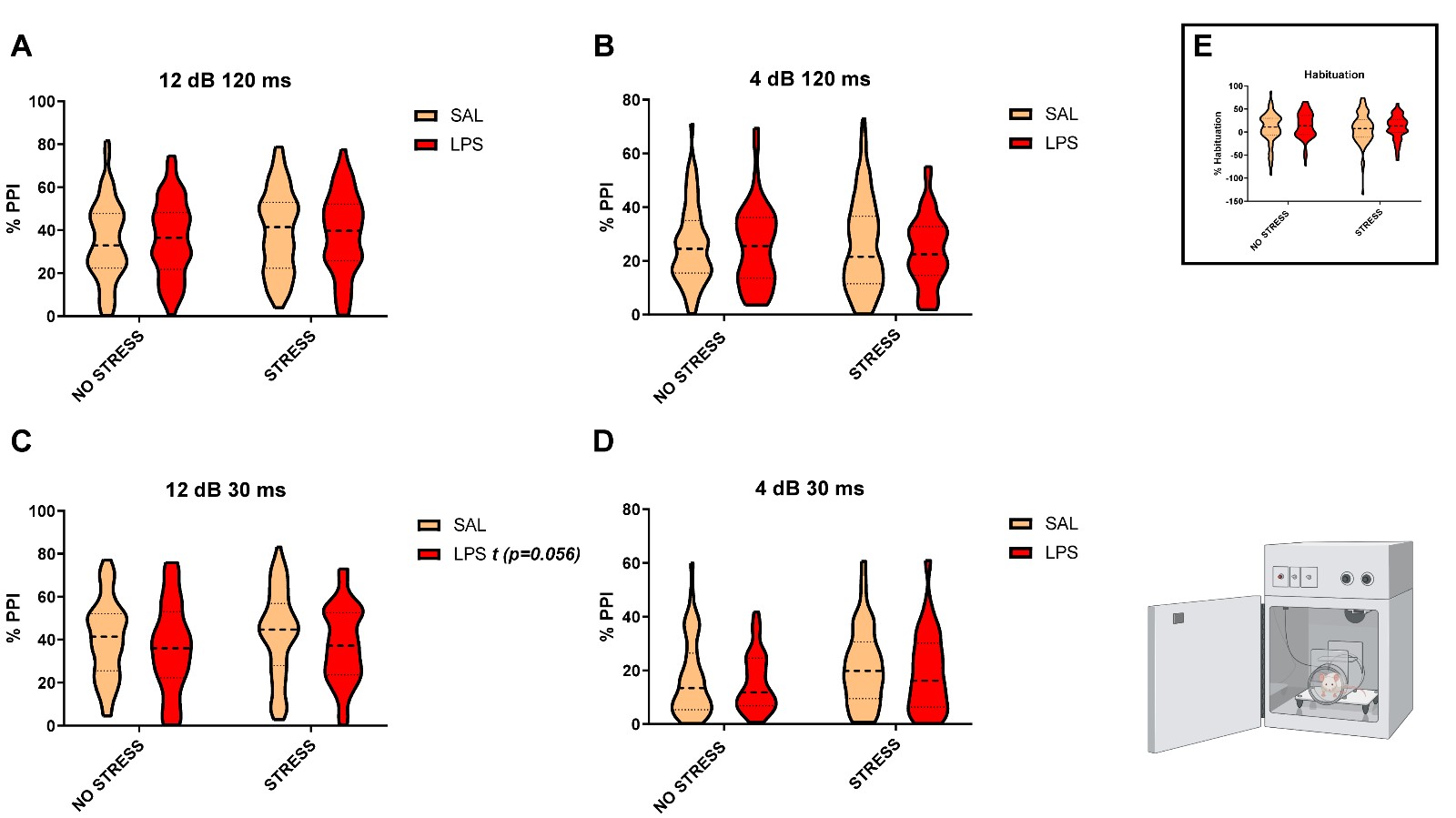


**Figure S1: Effects of prenatal LPS treatment or PUS in % PPI.** The figure shows % PPI at (A) 12 dB prepulse intensity and 120 ms interval (SAL+NS: n=82; SAL+S: n=82; LPS+NS: n=56; LPS+S: n=55); (B) 4 dB prepulse intensity and 120 ms interval (SAL+NS: n=69; SAL+S: n=69; LPS+NS: n=50; LPS+S: n=44); (C) 12 dB prepulse intensity and 30 ms interval (SAL+NS: n=80; SAL+S: n=83; LPS+NS: n=59; LPS+S: n=52). (D) 4 dB

prepulse intensity and 30 ms interval (SAL+NS: n=50; SAL+S: n=46; LPS+NS: n=33;

LPS+S: n=27). Note that animals with negative values (suggestive of prepulse facilitation

-PPF-) were discarded, and hence the difference in sample size across prepulse intensities (lower prepulse intensities tend to yield negative PPI values indicating PPF).


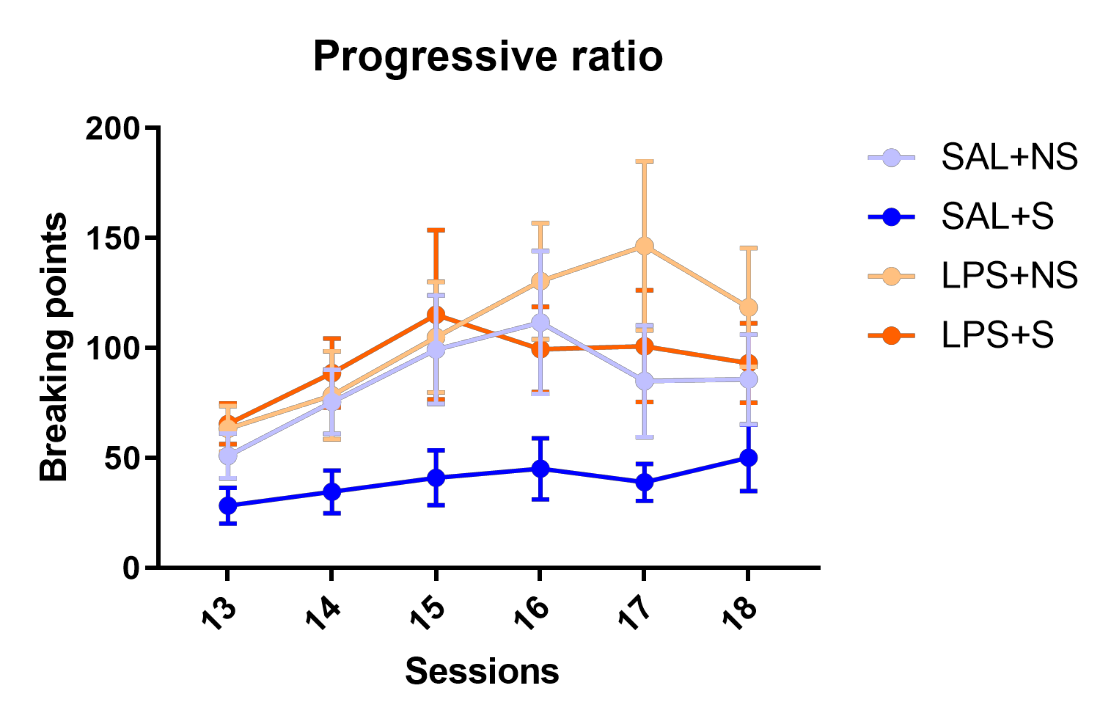


**Figure S2: Breaking points across the progressive ratio session:** Breaking points (defined as the last ordinal value of injection achieved before the rat failed to complete the requirement of an additional injection) across the progressive ration sessions. Rats showed a stable performance across sessions. No significant effects were observed as a consequence of MIA, PUS or interaction (SAL+NS: n=13; SAL+S: n=13; LPS+NS: n=13; LPS+S: n=15).


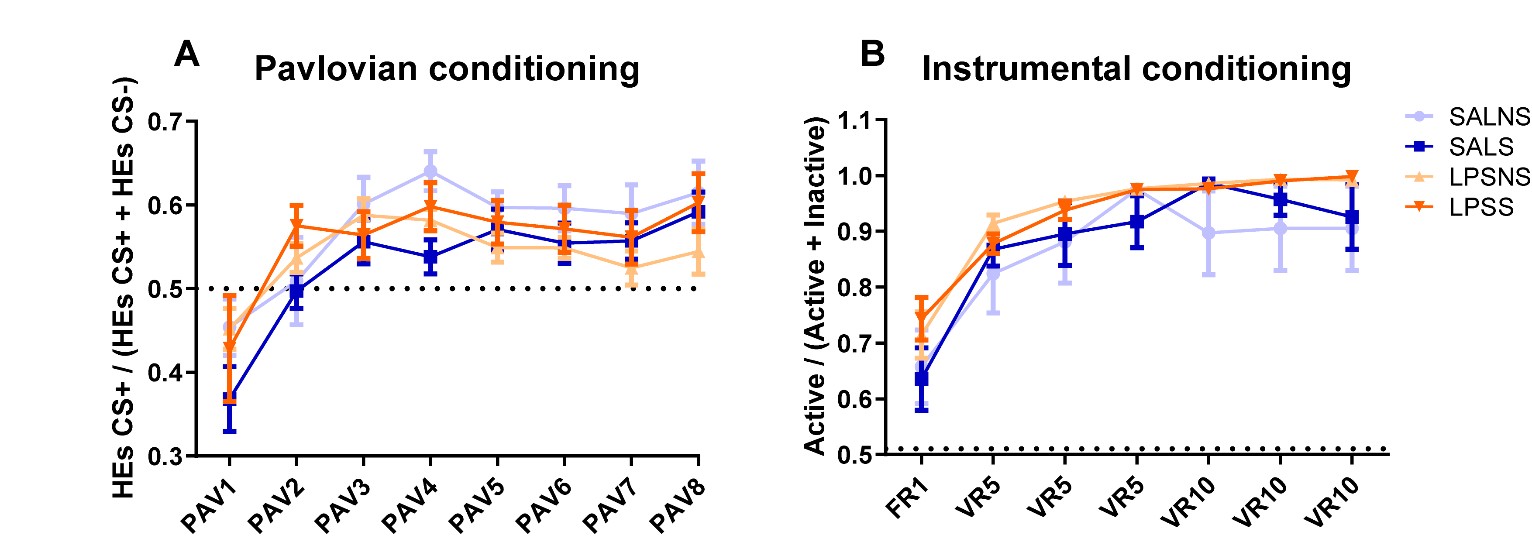


**Figure S3: Performance on Pavlovian and Instrumental Conditioning Tasks:** Ratio of head entries during the presence of the stimulus predictive of reward delivery (CS+) over the head entries during CS+ and the stimulus predictive of reward absence (CS+), across the eight conditioning sessions. Rats showed a stable performance across sessions. No significant effects were observed as a consequence of MIA, PUS or interaction (SAL+NS: n=13; SAL+S: n=17; LPS+NS: n=19; LPS+S: n=11).


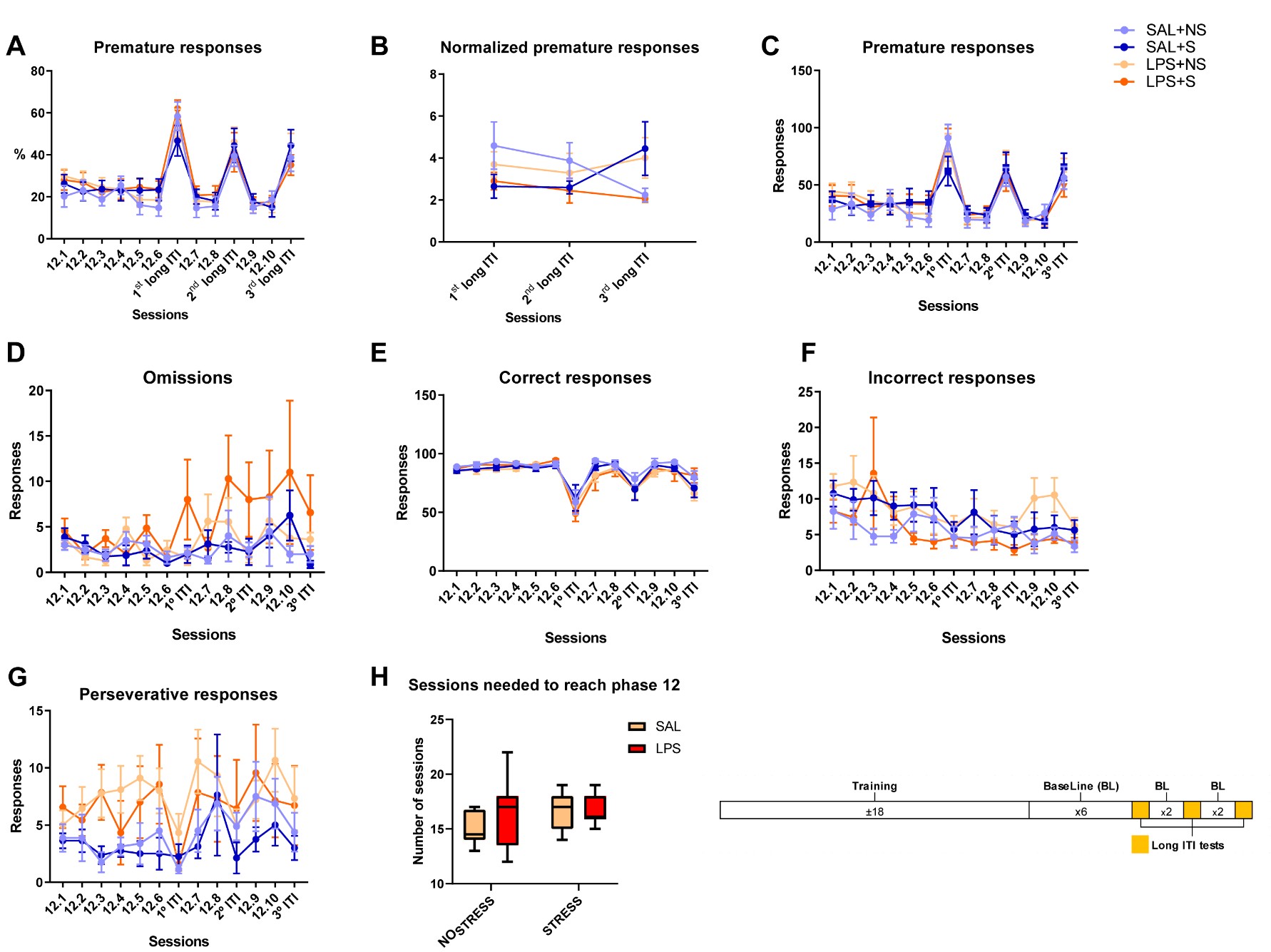


**Figure S4: Percentages of premature responses registered in the different sessions of the 2-CSRTT program.** The figure shows (A) the percentage of premature responses registered during the different sessions of the program [premature responses

/ (premature responses + correct responses + incorrect responses)], (B) the normalized percentage of premature responses registered during the three long ITI sessions of the program [% premature responses / mean % premature responses of the two previous sessions], (C) the number of premature responses, (D), number of omissions, (E) number of correct responses, (F) number of incorrect responses, (G) number of perseverative responses and (H) number of sessions needed to reach the final phase of training, phase 12, where the baseline was obtained. (SAL+NS: n=8; SAL+S: n=8; LPS+NS: n=9; LPS+S: n=7).


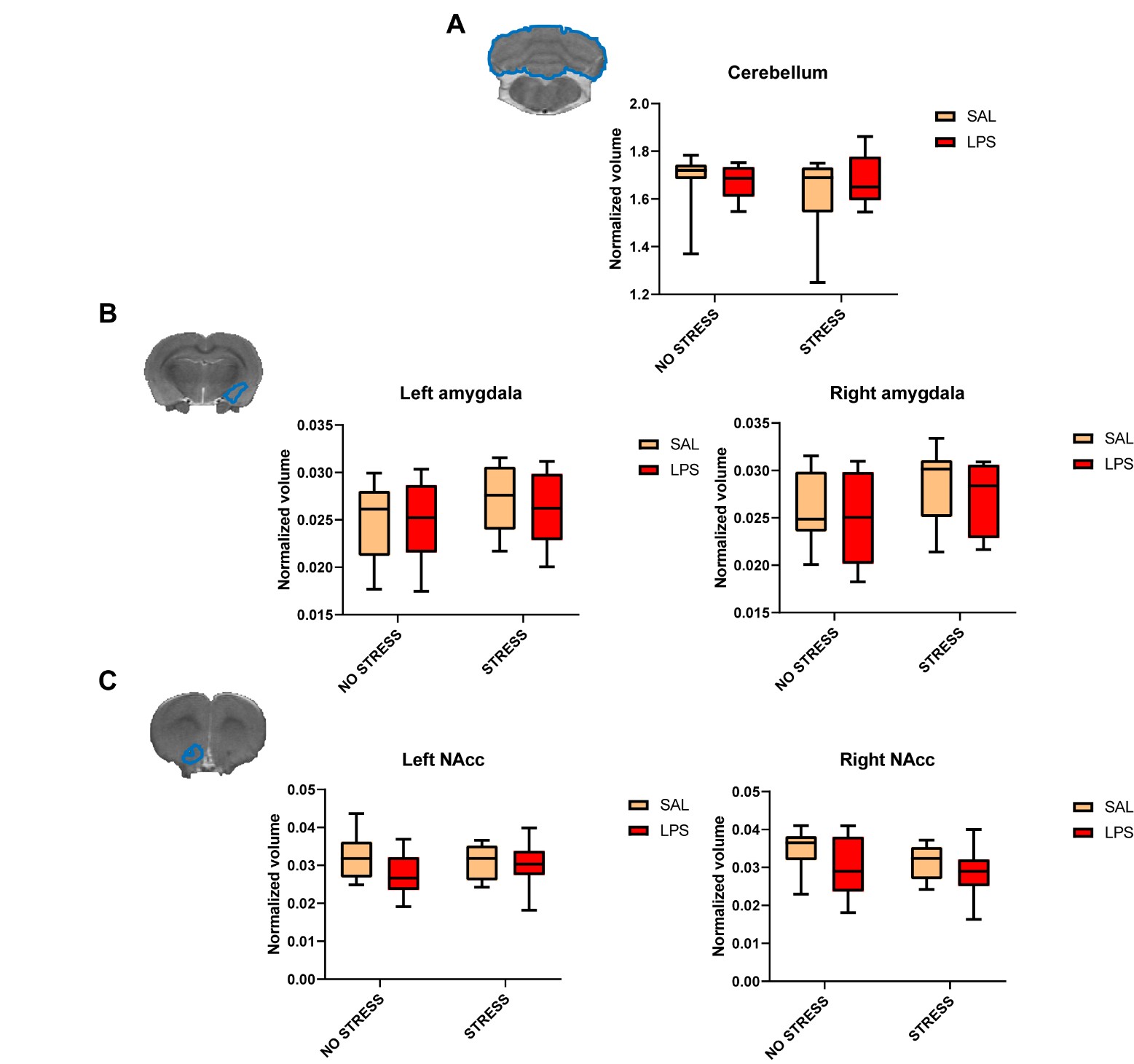


**Figure S5: MRI-assisted volumetry in additional brain areas.** The figure shows: (A) the normalized volume of the cerebellum, (B) the amygdala and (C) the NAcc. No significant effects were observed (SAL+NS: n=8; SAL+S: n=8; LPS+NS: n=8; LPS+S: n=7).


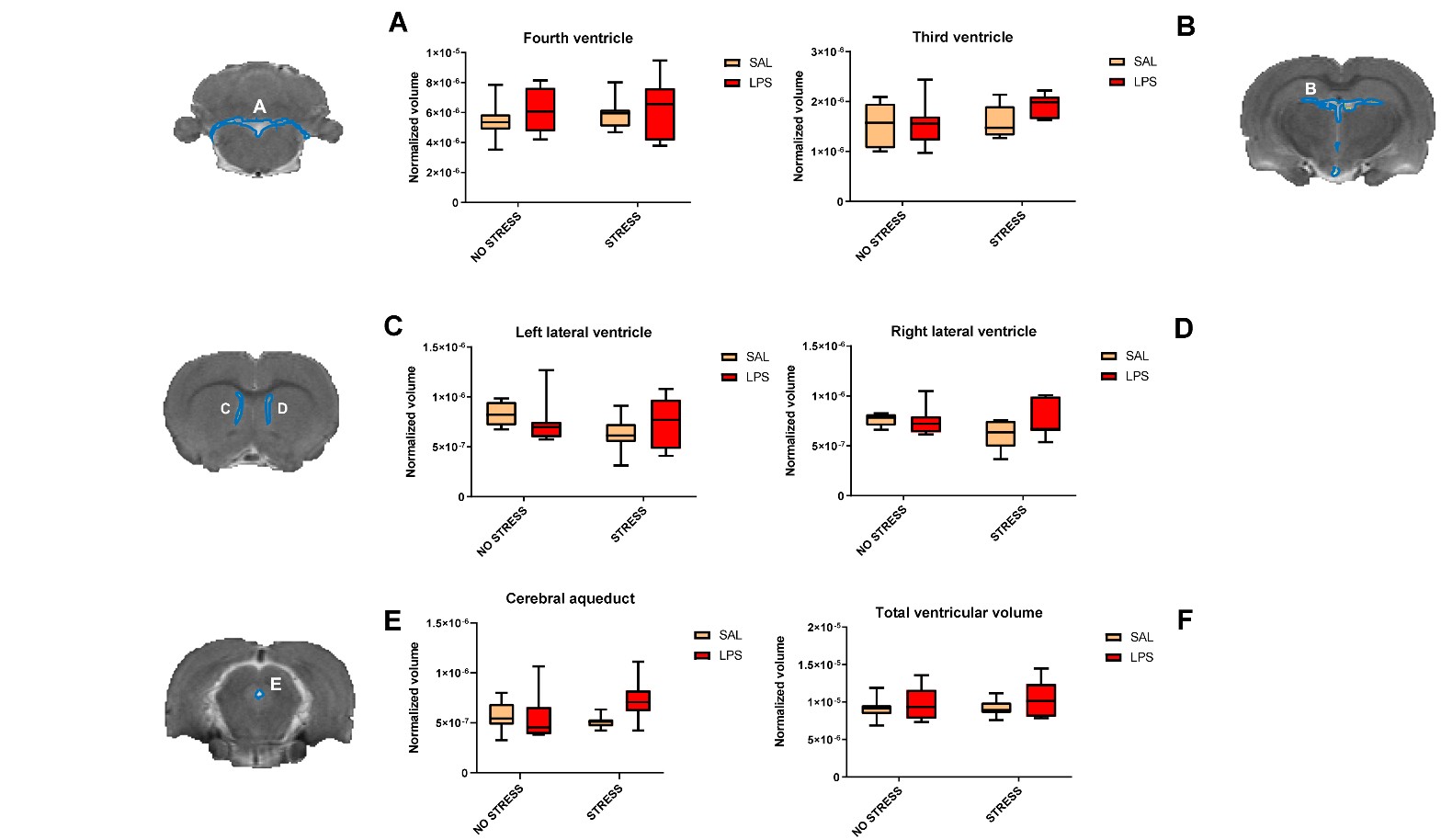


**Figure S6: MRI-assisted volumetry of the ventricular system.** The figure shows: (A) the normalized volume of the IV ventricle, (B) III ventricle and (C) the Aqueduct of Silvius or cerebral aqueduct. No significant effects were observed (SAL+NS: n=8; SAL+S: n=8; LPS+NS: n=8; LPS+S: n=7).


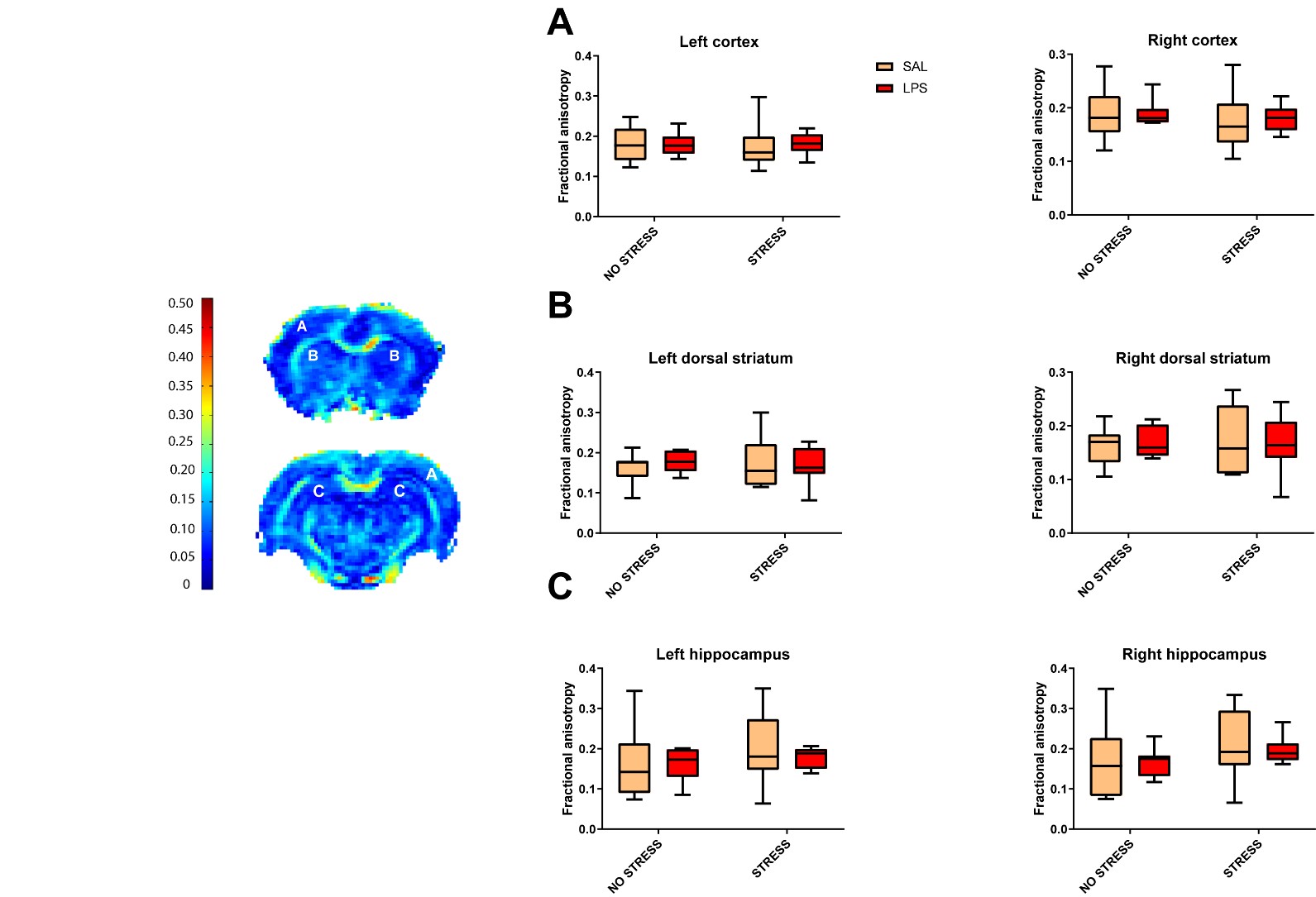


**Figure S7:** DTI FA analysis of specific brain structures. The figure shows the FA value of (A) the cortex, (B) the dorsal striatum and (C) the hippocampus. No significant effects were observed (SAL+NS: n=8; SAL+S: n=8; LPS+NS: n=8; LPS+S: n=7).


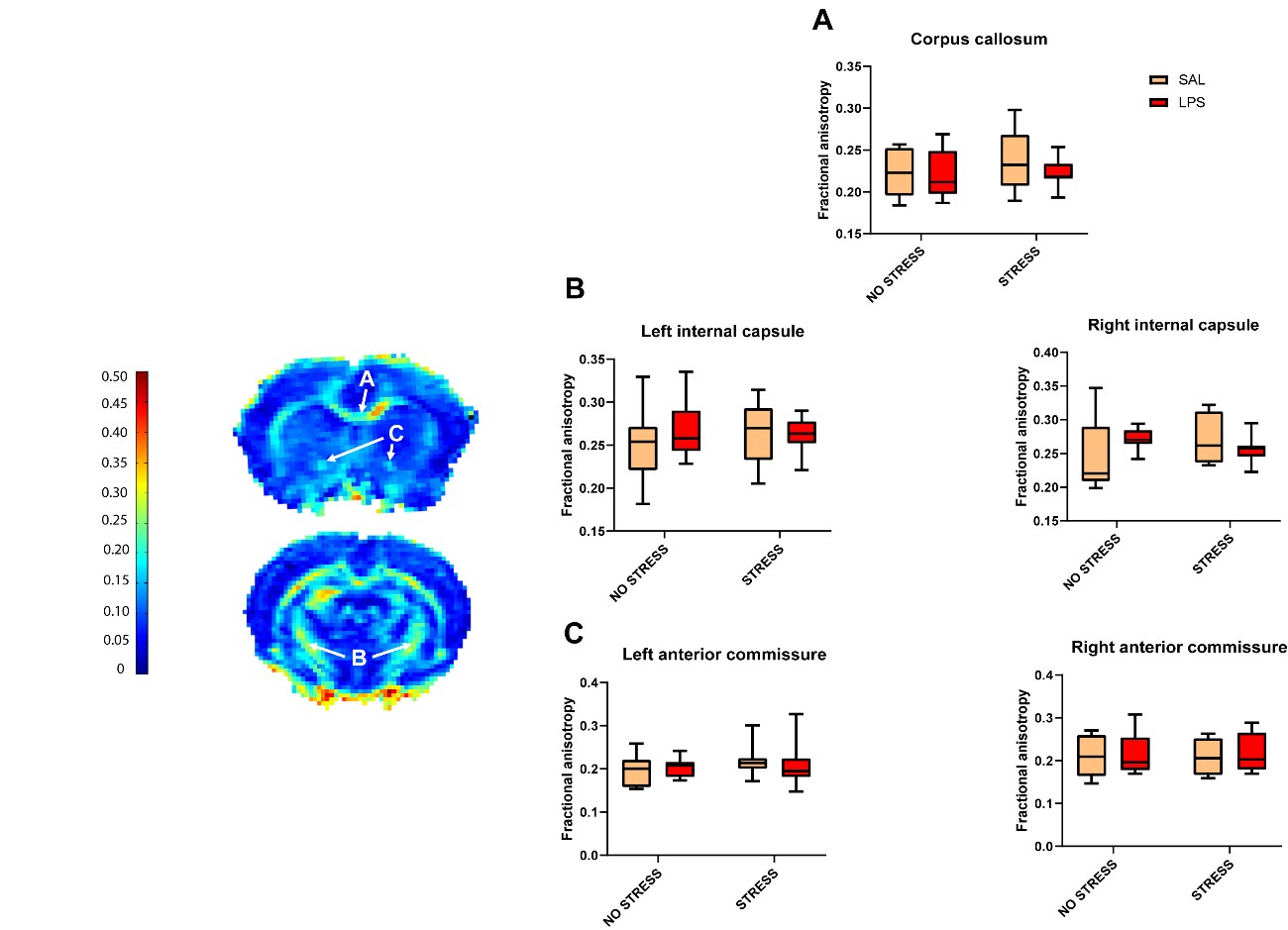


**Figure S8:** DTI FA analysis of specific brain tracts. The figure shows the FA value of (A) the corpus callosum, (B) the internal capsule and (C) the anterior commissure. No significant effects were observed (SAL+NS: n=8; SAL+S: n=8; LPS+NS: n=8; LPS+S: n=7).


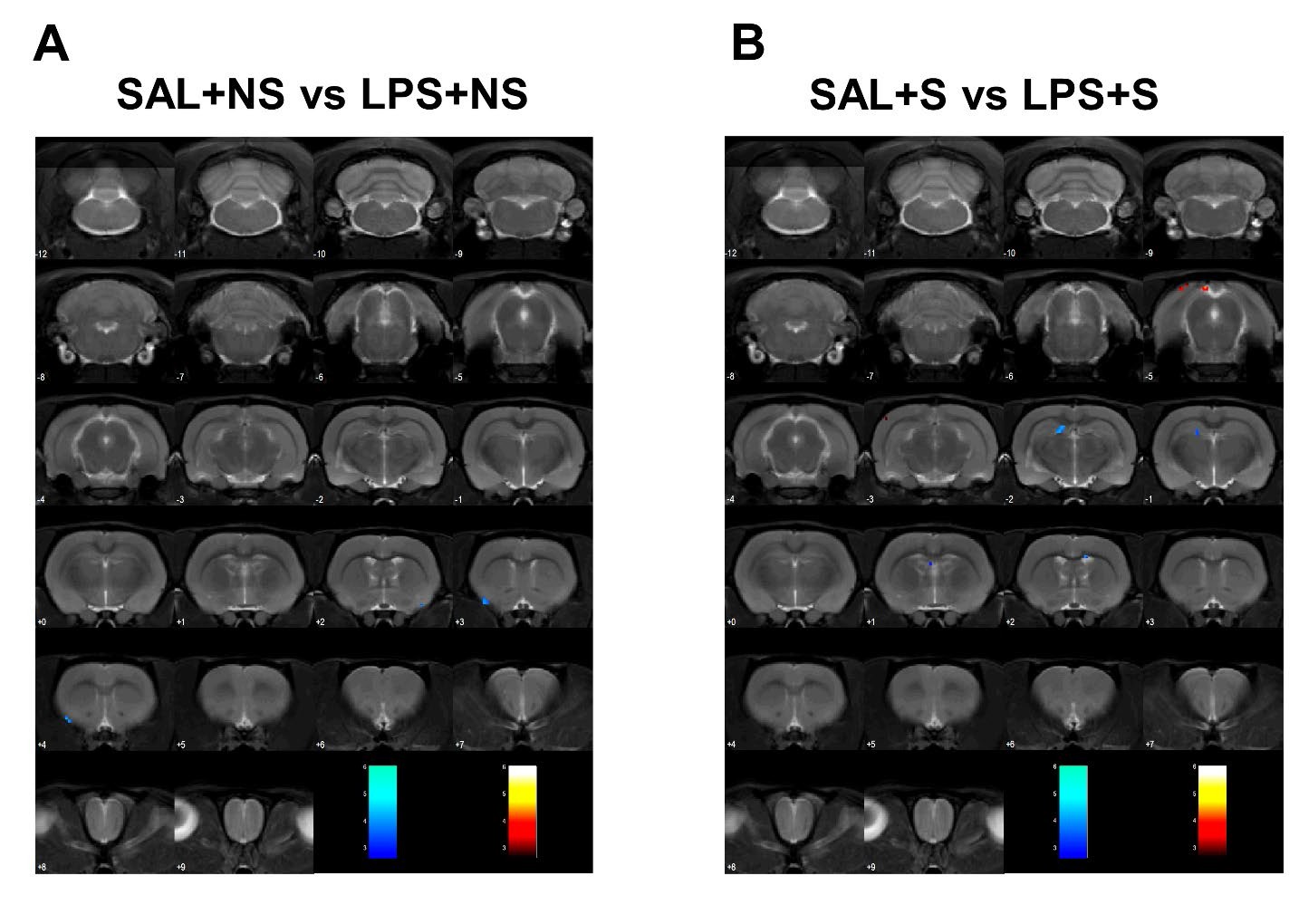


**Figure S9:** SPM analysis of PET data. No significant effects were observed as a result of MIA either in non-stressed or stressed animals (SAL+NS: n=5; SAL+S: n=5; LPS+NS: n=4; LPS+S: n=4). p<0.01 uncorrected, k>50 voxels

**NAcc**

**SAL+S vs LPS+S**


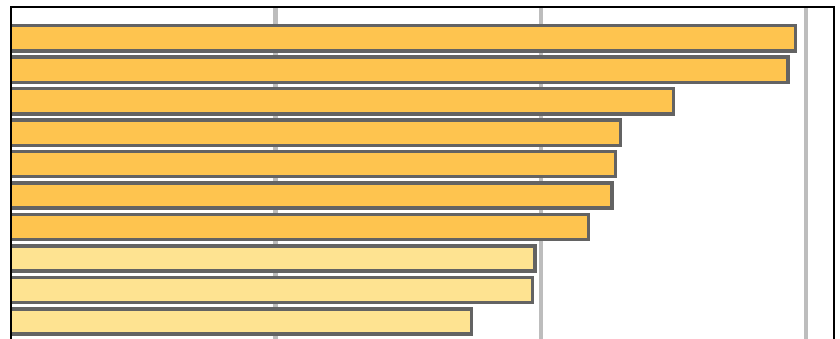

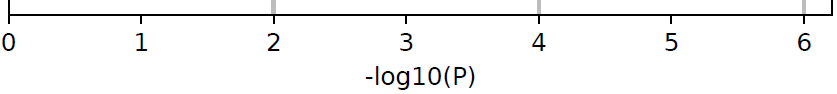


**Enrichment**

**Top 10 GO Biological processes**


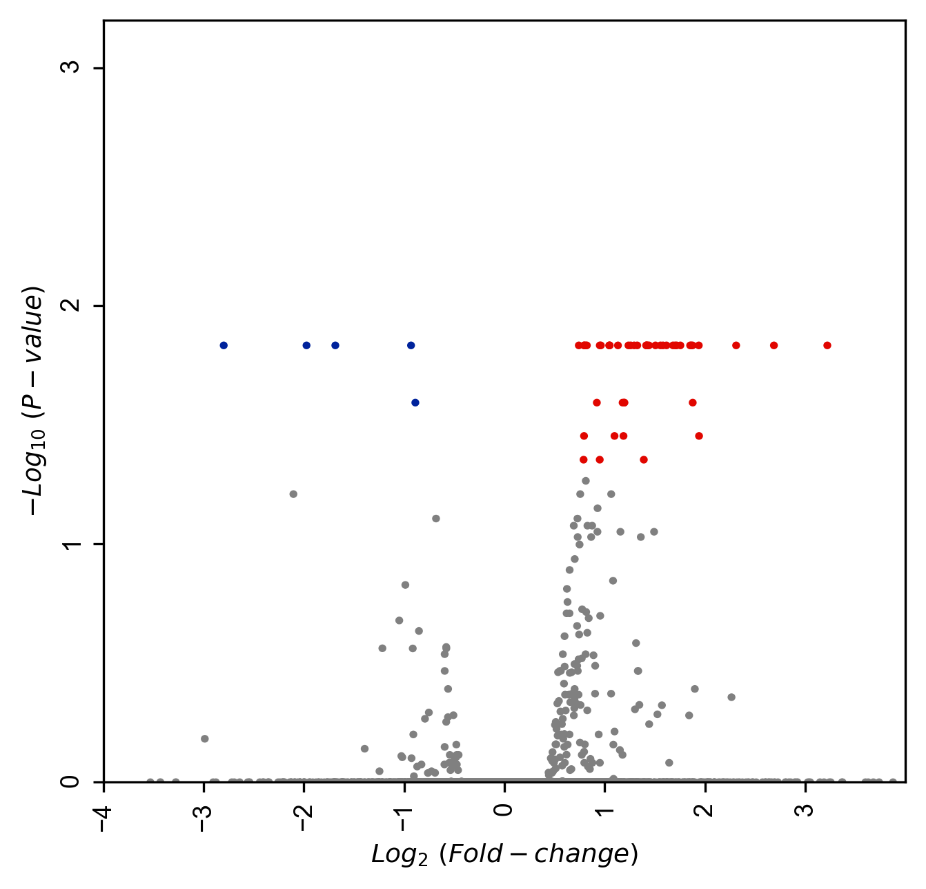

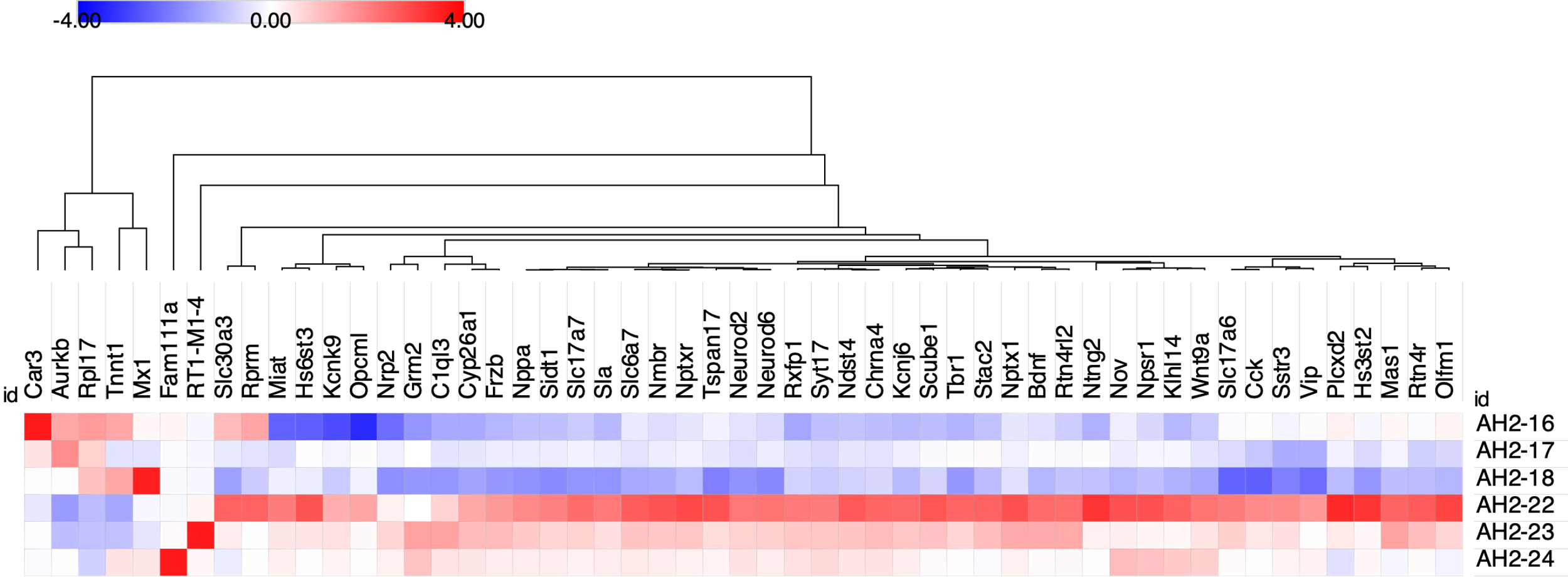

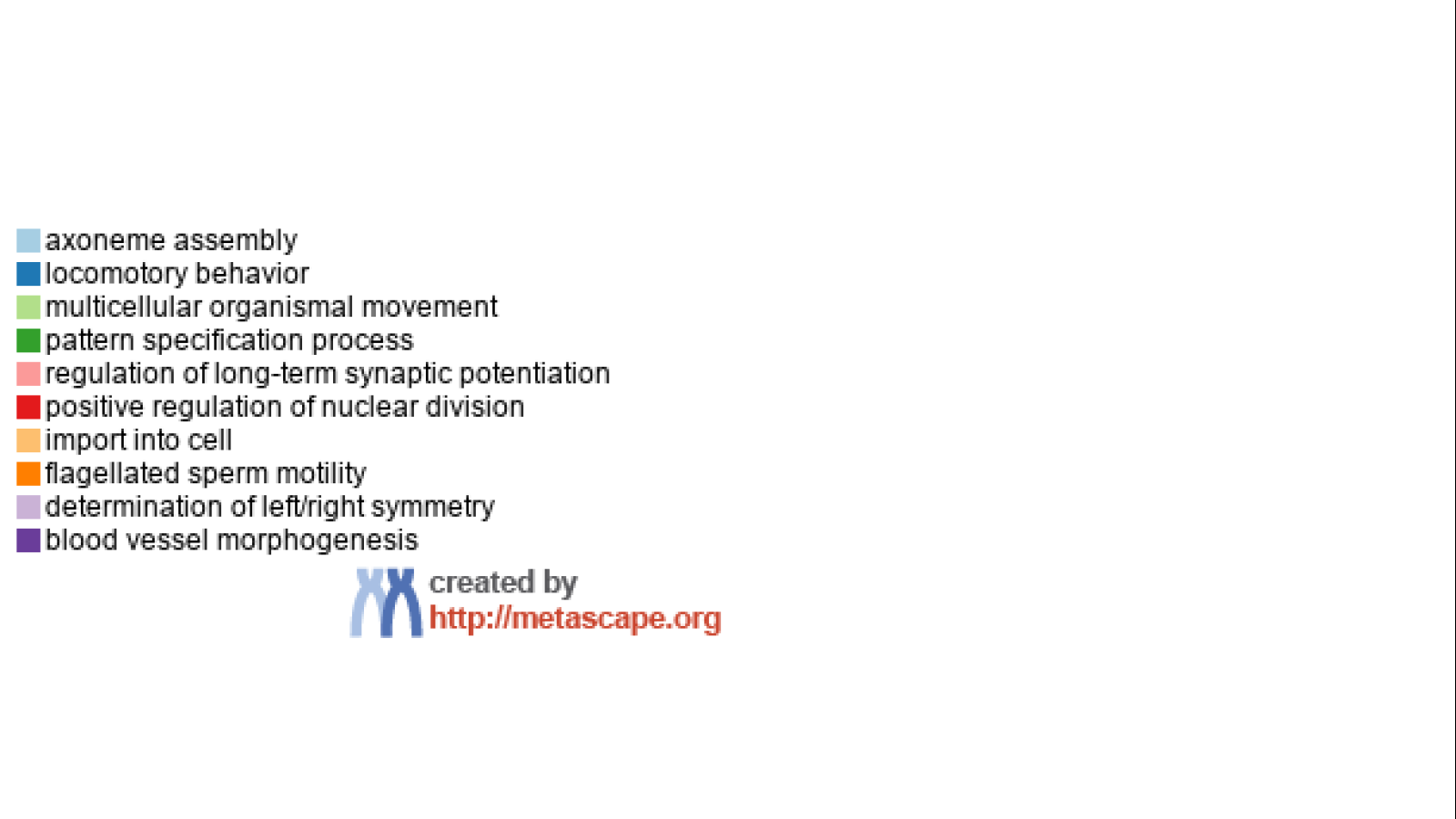


**https://software.broadinstitute.org/morpheus**

**SAL+S**

**LPS+S**


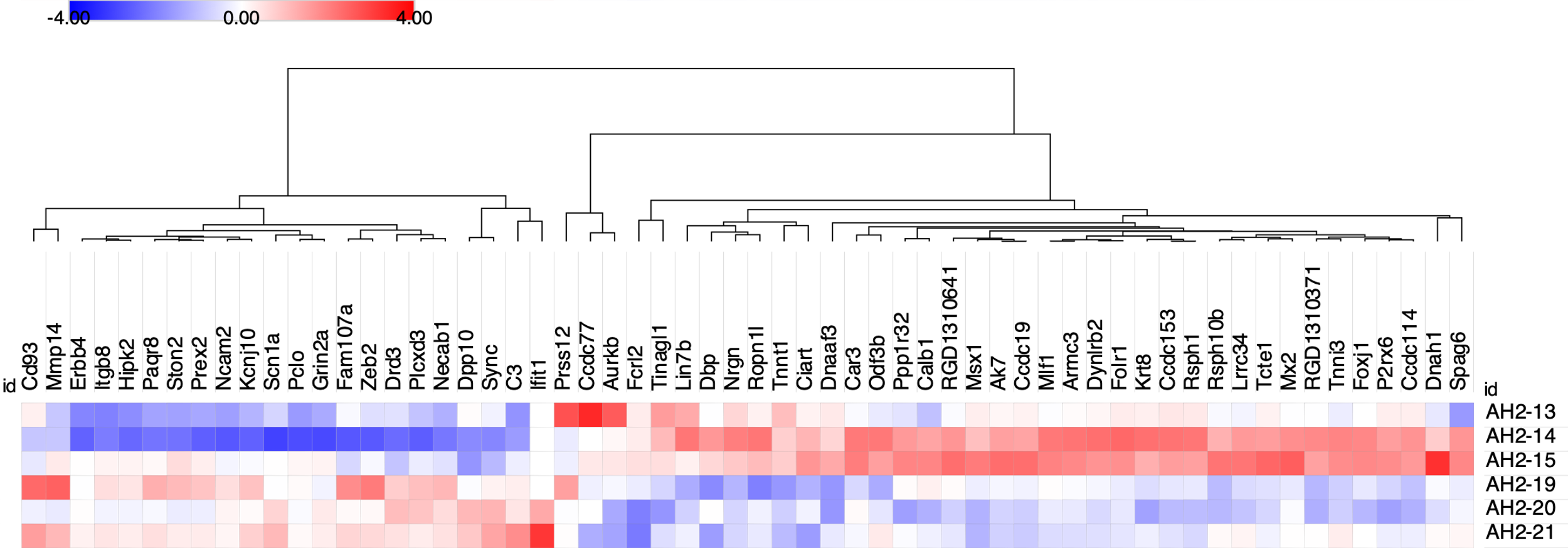


Fold-change

***Mx1***

***Opcml***

***Nrp2***

***Kcnk9***

***Grm2***

***Rxfp1***

***Stac2***

***Vip***

***Mas1***

***Ndst4***

***Hs6st3***

***Neurod6***

***Neurod2***

***Slc17a7***

***Rtn4rl2***

**Figure S10: RNAseq analysis in the NAcc for the SAL+S vs LPS+S comparison.** The figure shows a volcano-plot distribution of up- and down-regulated genes according to their p-value and fold-change, the top 10 GO biological processes in which they are involved, associated bar charts of enriched terms coloured by p-value (where the terms containing more genes show more significant p-value) and a heatmap showing fold-change values of differentially expressed genes in the NAcc for the SAL+S vs LPS+S comparison. (SAL+S= 3; LPS+S=3).


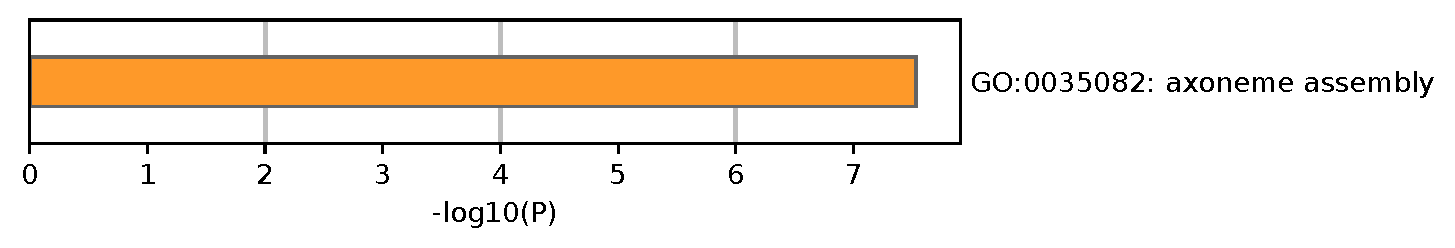

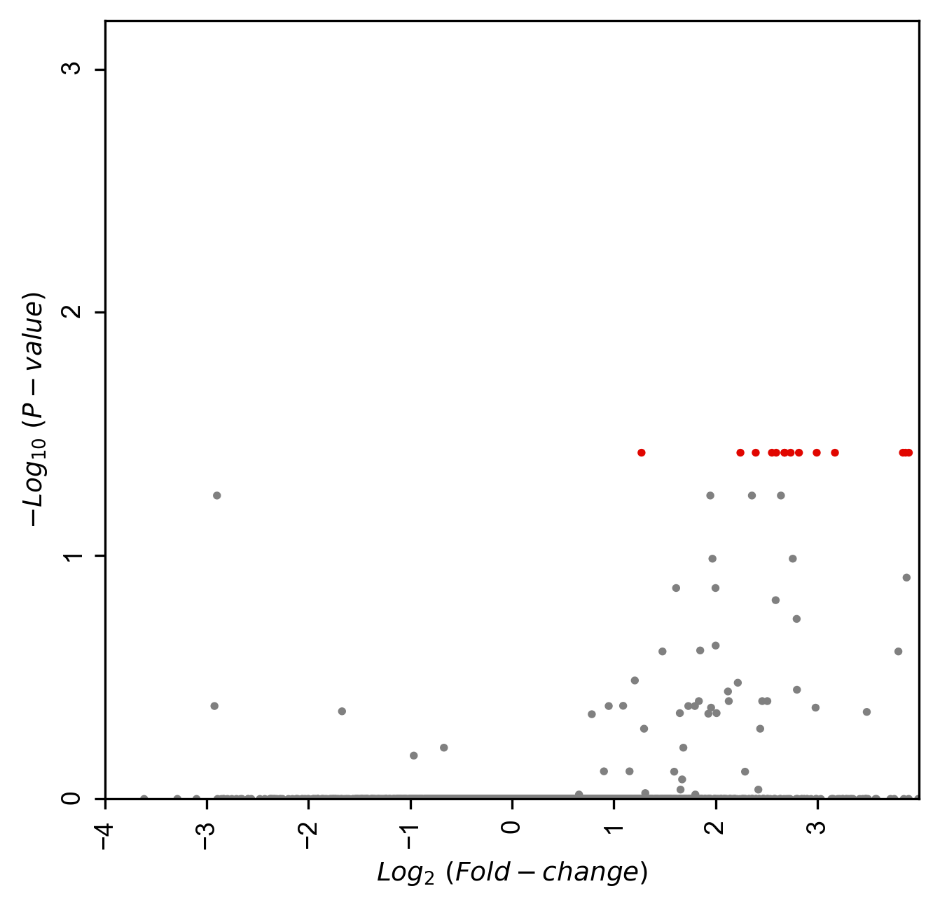


**Enrichment**


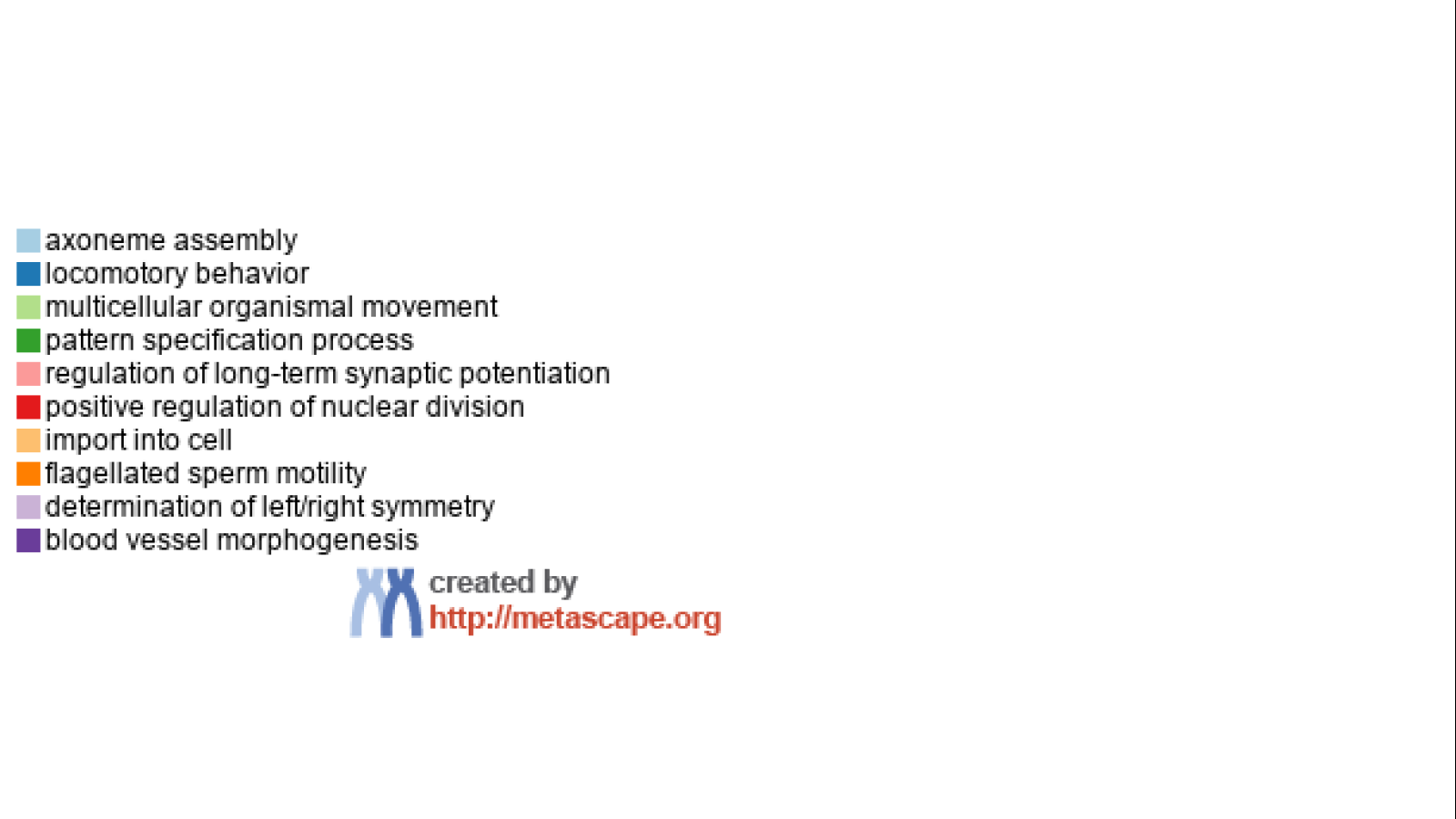


**https://software.broadinstitute.org/morpheus**

**Top 10 GO Biological processes**

**DLS**

**LPS+NS vs LPS+S**


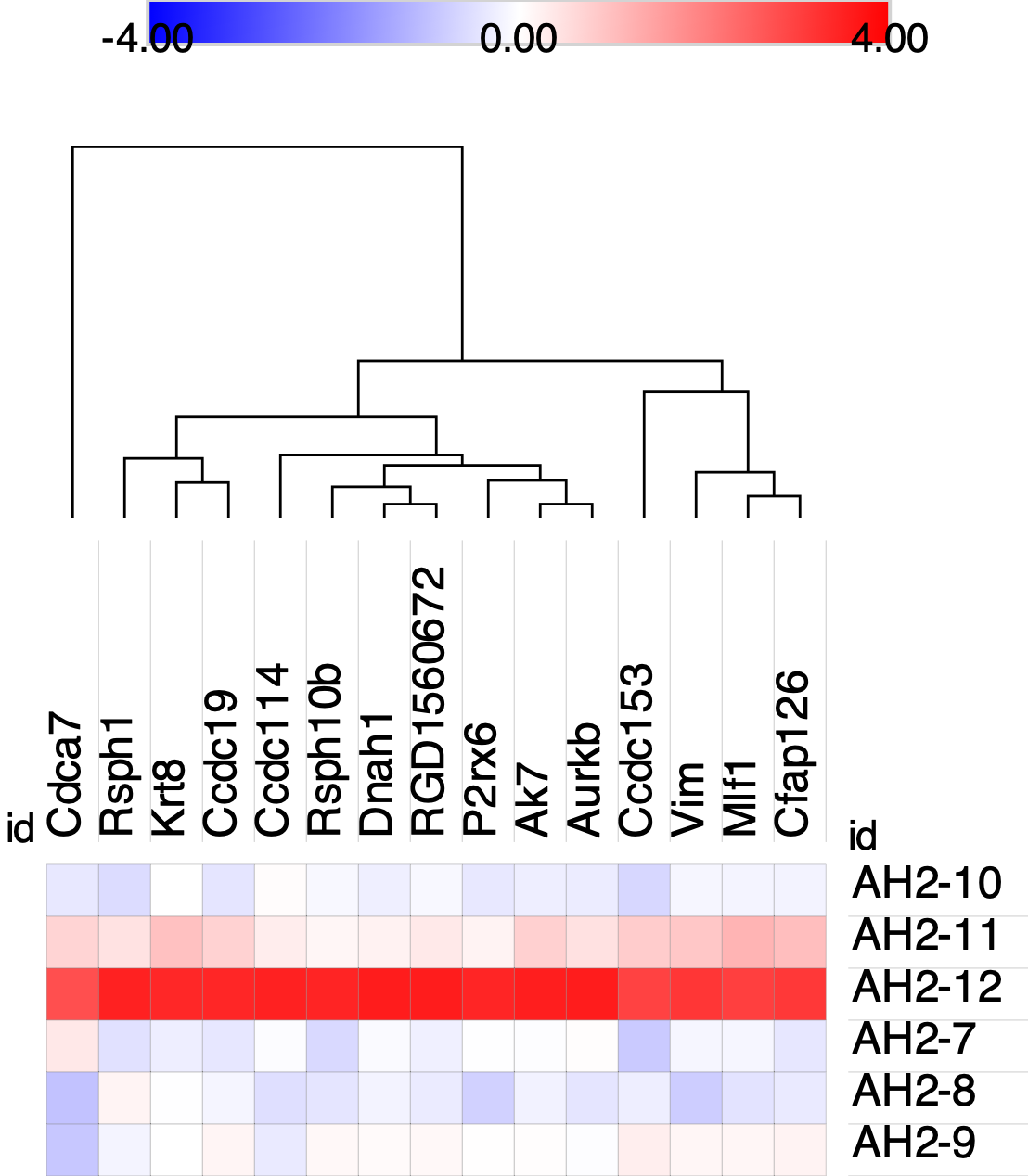


**LPS+S**

**LPS+NS**


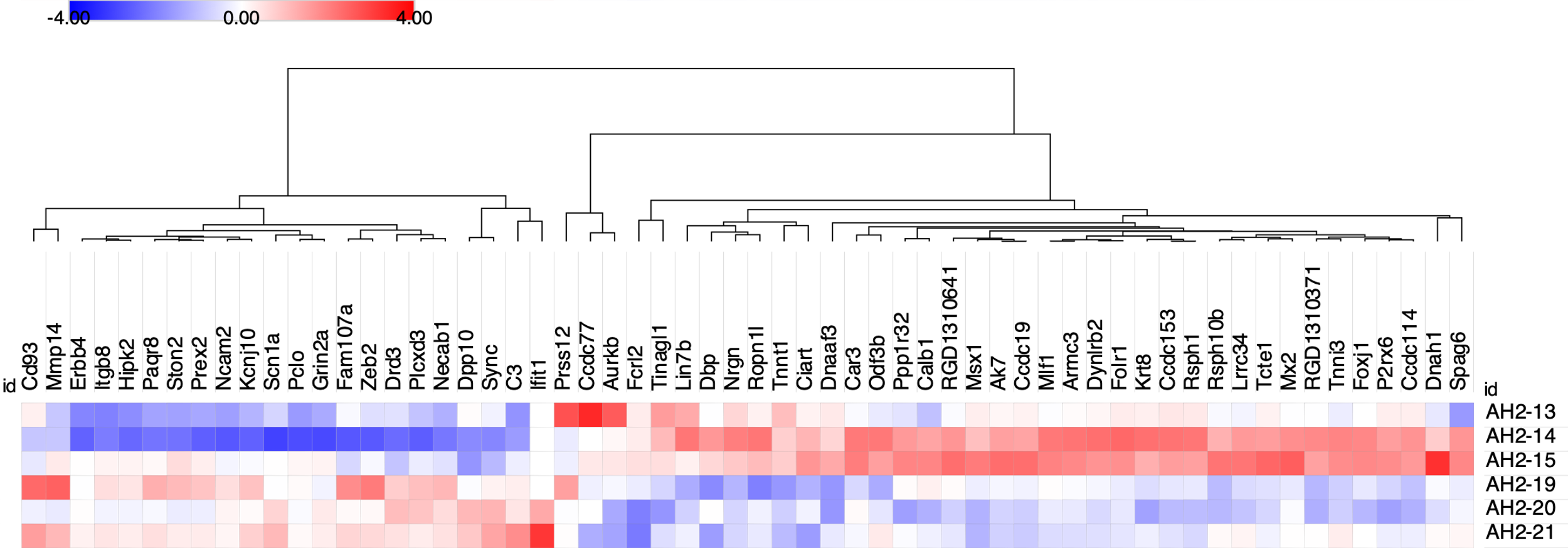


Fold-change

***Rsph1***

***Ccdc114***

***Ak7***

***Dnah1***

**Figure S11: RNAseq analysis in the dorsolateral striatum (DLS) for the SAL+NS vs LPS+S comparison.** The figure shows a volcano-plot distribution of up- and down-regulated genes according to their p-value and fold-change, the top 10 GO biological processes in which they are involved, associated bar charts of enriched terms coloured by p-value (where the terms containing more genes show more significant p-value) and a heatmap showing fold-change values of differentially expressed genes in the DLS for the SAL+NS vs LPS+S comparison. (SAL+NS= 3; LPS+S=3).


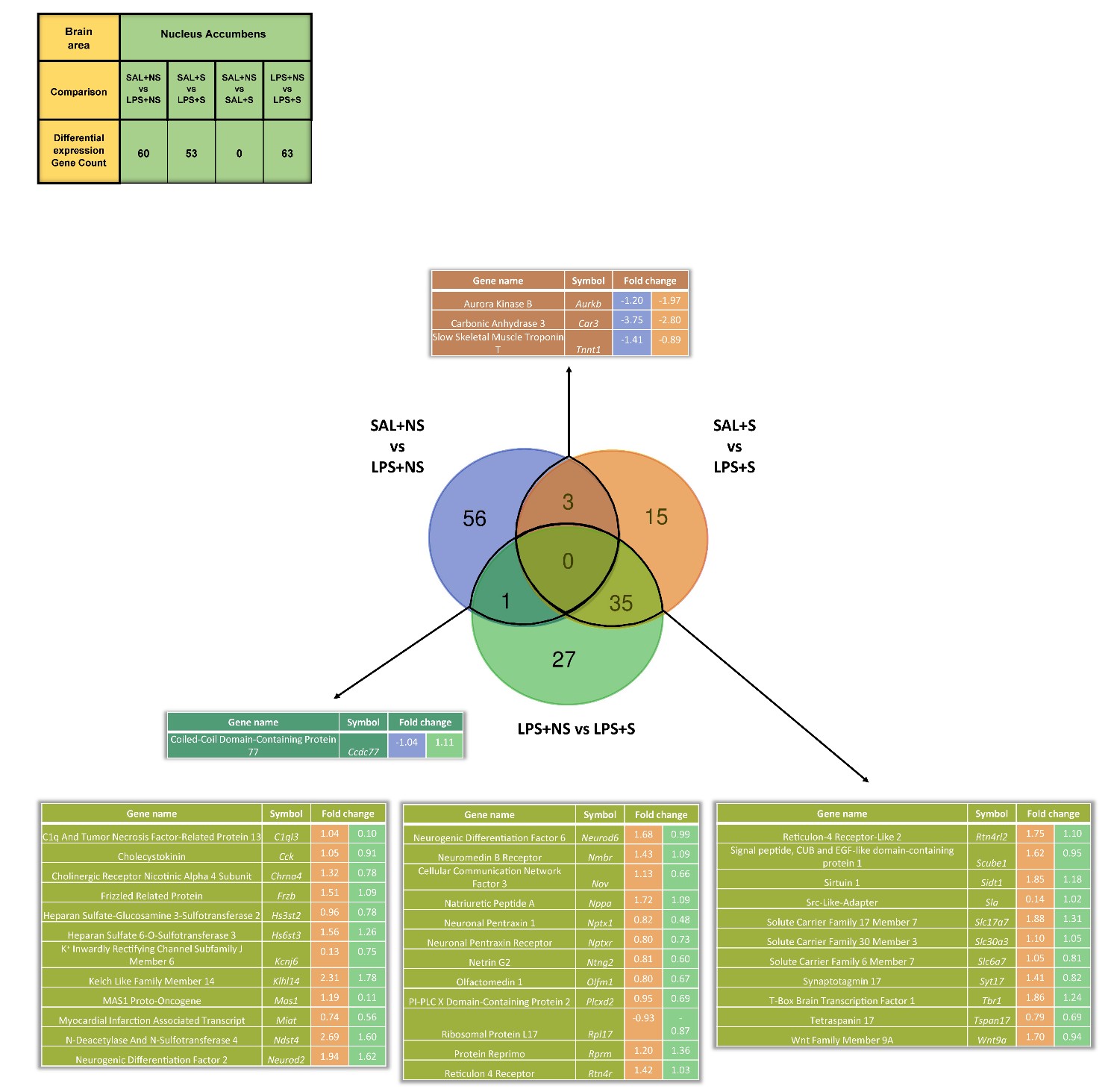


**Figure S12: Number of DEG per condition and DEG overlap between experimental groups in the NAcc.** The figure shows the number of DEG in each of the four groups and the overlap between the different conditions with tables showing the DEG for each overlap with the associated fold-change. Each colour represents the specific overlapping DEG subgroup. (SAL+NS: n=3; LPS+NS: n=3; SAL+S= 3; LPS+S=3).


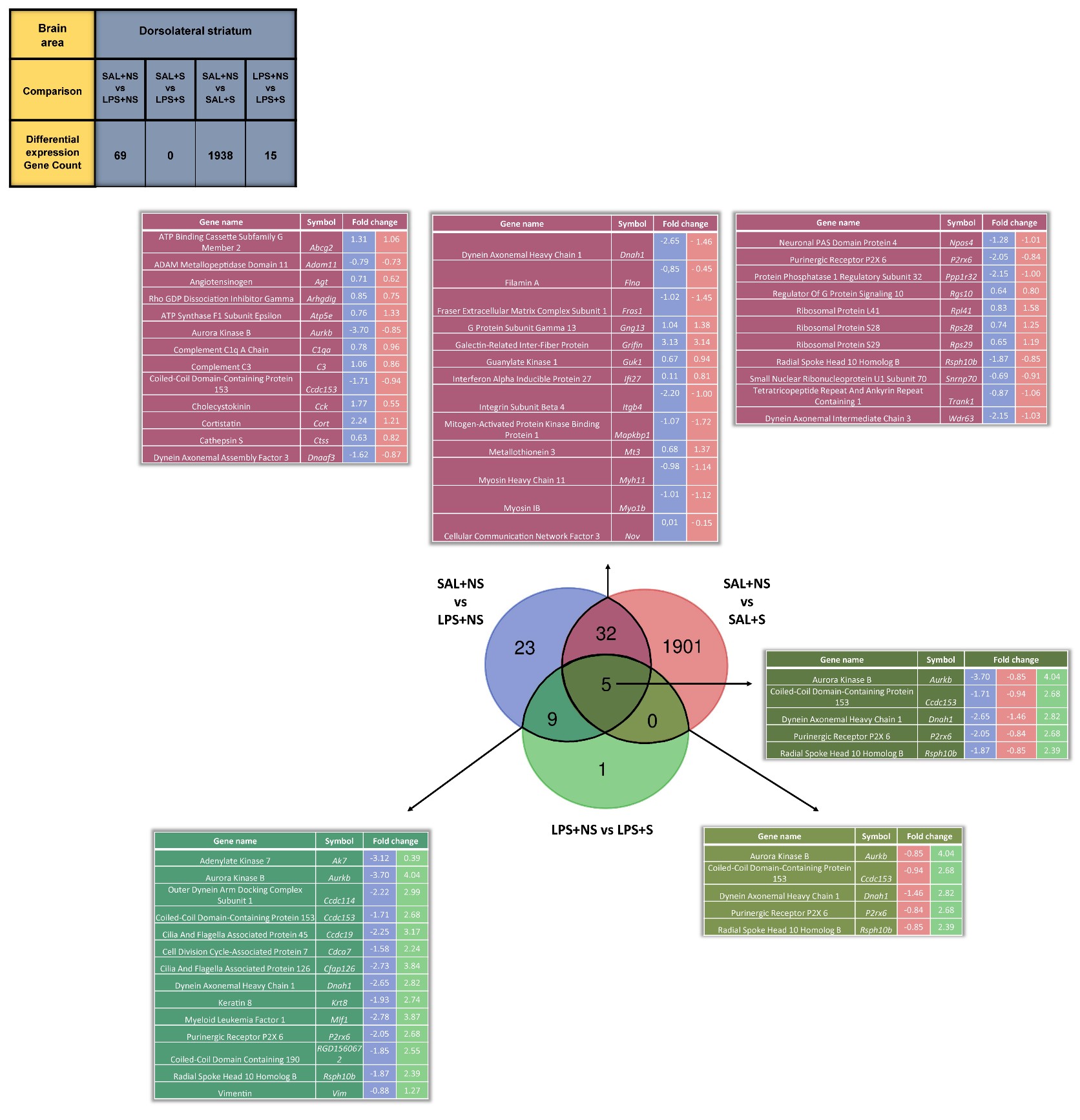


**Figure S13: Number of DEG per condition and DEG overlap between experimental groups in the DLS.** The figure shows the number of DEG in each of the four groups and the overlap between the different conditions with tables showing the DEG for each overlap with the associated fold-change. Each colour represents the specific overlapping DEG subgroup. (SAL+NS: n=3; LPS+NS: n=3; SAL+S= 3; LPS+S=3).

| **Global** |  | |
| --- | --- | --- |
| **PPI Condition** | **F-value and degrees of freedom** | **P-value** |
| **12 dB 120 ms** |  |  |
| **Prenatal immune activation** | **F (1, 271) = 0.019** | **0.891** |
| **Peripubertal unpredictable stress** | **F (1, 271) = 2.412** | **0.122** |
| **Interaction** | **F (1, 271) = 0.325** | **0.569** |
| **4 dB 120 ms** |  |  |
| **Prenatal immune activation** | **F (1, 228) = 0.423** | **0.516** |
| **Peripubertal unpredictable stress** | **F (1, 228) = 0.160** | **0.689** |
| **Interaction** | **F (1, 228) = 0.279** | **0.598** |
| **12 dB 30 ms** |  |  |
| **Prenatal immune activation** | **F (1, 270) = 3.678** | ***0.056*** |
| **Peripubertal unpredictable stress** | **F (1, 270) = 0.514** | **0.474** |
| **Interaction** | **F (1, 270) = 0.068** | **0.795** |
| **4 dB 30 ms** |  |  |
| **Prenatal immune activation** | **F (1, 152) = 0.658** | **0.418** |
| **Peripubertal unpredictable stress** | **F (1, 152) = 3.195** | ***0.076*** |
| **Interaction** | **F (1, 152) = 0.001** | **0.972** |
| **Habituation to main stimulus** |  |  |
| **Prenatal immune activation** | **F (1, 282) = 0.707** | **0.401** |
| **Peripubertal unpredictable stress** | **F (1, 282) = 0.176** | **0.675** |
| **Interaction** | **F (1, 282) = 0.576** | **0.447** |

###

### Table S1: F and p values of the ANOVAs used in the PPI experiment.

###
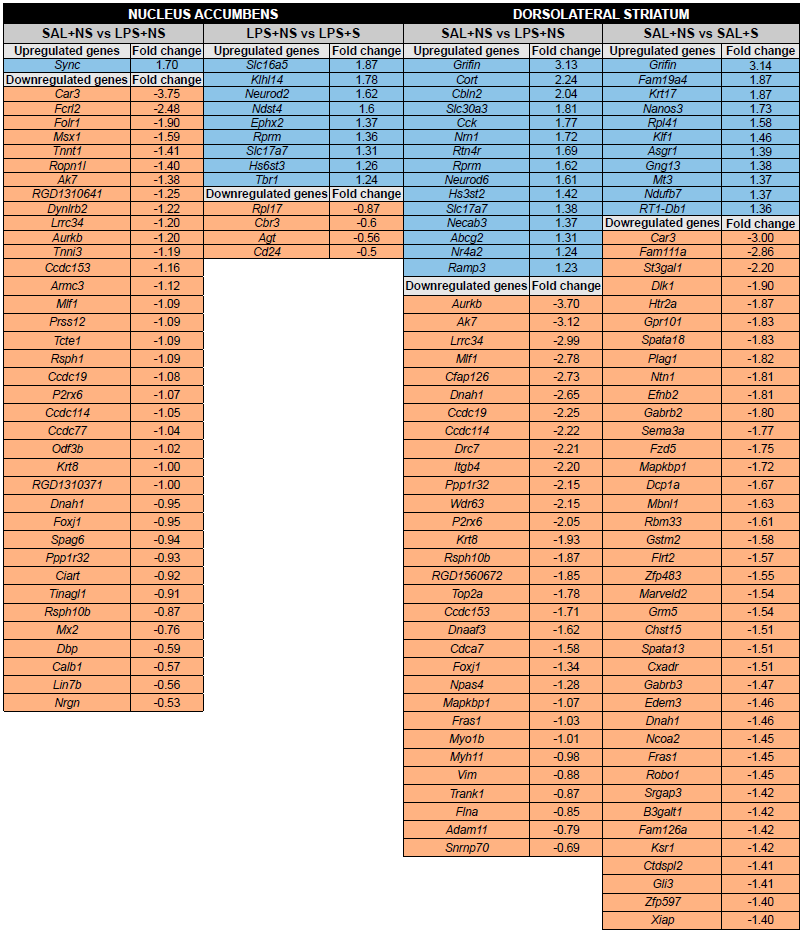
Table S2: Differentially expressed genes per condition and brain structure and associated fold-change.

# References

1 Boksa P. Effects of prenatal infection on brain development and behavior: a review of findings from animal models. *Brain Behav Immun* 2010; **24**: 881–897.

2 Fortier ME, Luheshi GN, Boksa P. Effects of prenatal infection on prepulse inhibition in the rat depend on the nature of the infectious agent and the stage of pregnancy. *Behav Brain Res* 2007; **181**: 270–277.

3 Wischhof L, Irrsack E, Osorio C, Koch M. Prenatal LPS-exposure - a neurodevelopmental rat model of schizophrenia - differentially affects cognitive functions, myelination and parvalbumin expression in male and female offspring. *Prog Neuropsychopharmacol Biol Psychiatry* 2015; **57**: 17–30.

4 Santos-Toscano R, Borcel É, Ucha M, Orihuel J, Capellán R, Roura-Martínez D *et al.* Unaltered cocaine self-administration in the prenatal LPS rat model of schizophrenia. *Prog Neuro-Psychopharmacology Biol Psychiatry* 2016; **69**. doi:10.1016/j.pnpbp.2016.04.008.

5 Giovanoli S, Engler H, Engler A, Richetto J, Voget M, Willi R *et al.* Stress in puberty unmasks latent neuropathological consequences of prenatal immune activation in mice. *Science* 2013; **339**: 1095–9.

6 Bari A, Dalley JW, Robbins TW. The application of the 5-choice serial reaction time task for the assessment of visual attentional processes and impulse control in rats. *Nat Protoc* 2008; **3**: 759–67.

7 Belin D, Mar AC, Dalley JW, Robbins TW, Everitt BJ, Molander AC *et al.* High impulsivity predicts the switch to compulsive cocaine-taking. *Science (80- )* 2008; **320**: 1352–1355.

8 Provencher SW. Automatic quantitation of localized in vivo1H spectra with LCModel. *NMR Biomed* 2001; **14**: 260–264.

9 Casquero-Veiga M, Garcia-Garcõa D, Pascau J, Desco M, Soto-Montenegro ML. Stimulating the nucleus accumbens in obesity: A positron emission tomography study after deep brain stimulation in a rodent model. *PLoS One* 2018; **13**. doi:10.1371/journal.pone.0204740.

10 Gasull-Camós J, Soto-Montenegro ML, Casquero-Veiga M, Desco M, Artigas F, Castañé A. Differential patterns of subcortical activity evoked by glial GLT-1 blockade in prelimbic and infralimbic cortex: Relationship to antidepressant-like effects in rats. *Int J Neuropsychopharmacol* 2017; **20**: 988–993.

11 Paxinos G, Watson C. The rat brain in stereotaxic coordinates (6th ed.). *Acad Press* 2007.

12 De Peri L, Deste G, Vita A. Strucutural brain imaging at the onset of schizophrenia:What have we learned and what have we missed. *Psychiatry Res* 2021; **301**: 113962.

13 Kreitz S, Zambon A, Ronovsky M, Budinsky L, Helbich TH, Sideromenos S *et al.* Maternal immune activation during pregnancy impacts on brain structure and function in the adult offspring. *Brain Behav Immun* 2020; **83**: 56–67.

14 Fatemi SH, Reutiman TJ, Folsom TD, Huang H, Oishi K, Mori S *et al.* Maternal infection leads to abnormal gene regulation and brain atrophy in mouse offspring: Implications for genesis of neurodevelopmental disorders. *Schizophr Res* 2008; **99**: 56–70.

15 Li Q, Cheung C, Wei R, Hui ES, Feldon J, Meyer U *et al.* Prenatal immune challenge is an environmental risk factor for brain and behavior change relevant to schizophrenia: Evidence from MRI in a mouse model. *PLoS One* 2009; **4**. doi:10.1371/journal.pone.0006354.

16 Casquero-Veiga M, García-García D, MacDowell KS, Pérez-Caballero L, Torres-Sánchez S, Fraguas D *et al.* Risperidone administered during adolescence induced metabolic, anatomical and inflammatory/oxidative changes in adult brain: A PET and MRI study in the maternal immune stimulation animal model. *Eur Neuropsychopharmacol* 2019; **29**: 880–896.

17 Casquero-Veiga M, Romero-Miguel D, MacDowell KS, Torres-Sanchez S, Garcia-Partida JA, Lamanna-Rama N *et al.* Omega-3 fatty acids during adolescence prevent schizophrenia-related behavioural deficits: Neurophysiological evidences from the prenatal viral infection with PolyI:C. *Eur Neuropsychopharmacol* 2021; **46**: 14–27.

18 Lamb RJ, Jarbe TU. Multielemental stimulus control: effects of saccharin concentration on a discriminated morphine-saccharin taste aversion. *Exp Clin Psychopharmacol* 1997; **5**: 123–129.

19 Ball G, Srinivasan L, Aljabar P, Counsell SJ, Durighel G, Hajnal J V. *et al.* Development of cortical microstructure in the preterm human brain. *Proc Natl Acad Sci U S A* 2013; **110**: 9541–9546.

20 Li Y, Xie S, Liu B, Song M, Chen Y, Li P *et al.* Diffusion magnetic resonance imaging study of schizophrenia in the context of abnormal neurodevelopment using multiple site data in a chinese han population. *Transl Psychiatry* 2016; **6**. doi:10.1038/tp.2015.202.

21 Vigli D, Palombelli G, Fanelli S, Calamandrei G, Canese R, Mosca L *et al.* Maternal Immune Activation in Mice Only Partially Recapitulates the Autism Spectrum Disorders Symptomatology. *Neuroscience* 2020; **445**: 109–119.

22 Li Q, Leung YO, Zhou I, Ho LC, Kong W, Basil P *et al.* Dietary supplementation with n-3 fatty acids from weaning limits brain biochemistry and behavioural changes elicited by prenatal exposure to maternal inflammation in the mouse model. *Transl Psychiatry* 2015; **5**. doi:10.1038/tp.2015.126.

23 Vernon AC, So P-W, Lythgoe DJ, Chege W, Cooper JD, Williams SCR *et al.* Longitudinal in vivo maturational changes of metabolites in the prefrontal cortex of rats exposed to polyinosinic-polycytidylic acid in utero. *Eur Neuropsychopharmacol* 2015; **25**: 2210–20.

24 Romero-Miguel D, Casquero-Veiga M, MacDowell KS, Torres-Sanchez S, Garcia-Partida JA, Lamanna-Rama N *et al.* A Characterization of the Effects of Minocycline Treatment During Adolescence on Structural, Metabolic, and Oxidative Stress Parameters in a Maternal Immune Stimulation Model of Neurodevelopmental Brain Disorders. *Int J Neuropsychopharmacol* 2021; **24**: 734–748.

25 Hadar R, Soto-Montenegro ML, Götz T, Wieske F, Sohr R, Desco M *et al.* Using a maternal immune stimulation model of schizophrenia to study behavioral and neurobiological alterations over the developmental course. *Schizophr Res* 2015; **166**: 238–247.

26 Hadar R, Bikovski L, Soto-Montenegro ML, Schimke J, Maier P, Ewing S *et al.* Early neuromodulation prevents the development of brain and behavioral abnormalities in a rodent model of schizophrenia. *Mol Psychiatry* 2018; **23**: 943–951.

27 Brugger S, Davis JM, Leucht S, Stone JM. Proton Magnetic Resonance Spectroscopy and Illness Stage in Schizophrenia—A Systematic Review and Meta-Analysis. *Biol Psychiatry* 2011; **69**: 495–503.

28 Kraguljac NV, Reid M, White D, Jones R, den Hollander J, Lowman D *et al.* Neurometabolites in schizophrenia and bipolar disorder - A systematic review and meta-analysis. Psychiatry Res. - Neuroimaging. 2012; **203**: 111–125.

29 Capellán R, Moreno-Fernández M, Orihuel J, Roura-Martínez D, Ucha M, Ambrosio E *et al.* Ex vivo 1H-MRS brain metabolic profiling in a two-hit model of neurodevelopmental disorders: Prenatal immune activation and peripubertal stress. *Schizophr Res* 2022; **243**: 232–240.

30 Sinibaldi L, De Luca A, Bellacchio E, Conti E, Pasini A, Paloscia C *et al.* Mutations of the nogo-66 receptor (Rtn4r) gene in schizophrenia. *Hum Mutat* 2004; **24**: 534–535.

31 Perlstein MD, Chohan MR, Coman IL, Antshel KM, Fremont WP, Gnirke MH *et al.* White matter abnormalities in 22q11.2 deletion syndrome: Preliminary associations with the Nogo-66 receptor gene and symptoms of psychosis. *Schizophr Res* 2014; **152**: 117–123.

32 Thompson CA, Karelis J, Middleton FA, Gentile K, Coman IL, Radoeva PD *et al.* Associations between neurodevelopmental genes, neuroanatomy, and ultra high risk symptoms of psychosis in 22q11.2 deletion syndrome. *Am J Med Genet Part B Neuropsychiatr Genet* 2017; **174**: 295–314.

33 Li J, Cai T, Jiang Y, Chen H, He X, Chen C *et al.* Genes with de novo mutations are shared by four neuropsychiatric disorders discovered from NPdenovo database. *Mol Psychiatry* 2016; **21**: 290–297.

34 Nambot S, Faivre L, Mirzaa G, Thevenon J, Bruel AL, Mosca-Boidron AL *et al.* De novo TBR1 variants cause a neurocognitive phenotype with ID and autistic traits: report of 25 new individuals and review of the literature. *Eur J Hum Genet* 2020; **28**: 770–782.

35 Di Carlo P, Punzi G, Ursini G. Brain-derived neurotrophic factor and schizophrenia. Psychiatr. Genet. 2020; **29**: 200–210.

36 Armeanu R, Mokkonen M, Crespi B. Meta-Analysis of BDNF Levels in Autism. *Cell Mol Neurobiol* 2017; **37**: 949–954.

37 Oni-Orisan A, Kristiansen L V., Haroutunian V, Meador-Woodruff JH, McCullumsmith RE. Altered Vesicular Glutamate Transporter Expression in the Anterior Cingulate Cortex in Schizophrenia. *Biol Psychiatry* 2008; **63**: 766–775.

38 Piontkewitz Y, Arad M, Weiner I. Abnormal trajectories of neurodevelopment and behavior following in utero insult in the rat. *Biol Psychiatry* 2011; **70**: 842–51.

39 McCutcheon RA, Abi-Dargham A, Howes OD. Schizophrenia, Dopamine and the Striatum: From Biology to Symptoms. *Trends Neurosci* 2019; **42**: 205–220.

40 Cáceda R, Kinkead B, Nemeroff CB. Involvement of Neuropeptide Systems in Schizophrenia: Human Studies. Int. Rev. Neurobiol. 2007; **78**: 327–376.

41 Ballaz SJ, Bourin M. Cholecystokinin-mediated Neuromodulation of Anxiety and Schizophrenia: A “Dimmer-Switch” Hypothesis. *Curr Neuropharmacol* 2020; **18**. doi:10.2174/1570159x18666201113145143.

42 Huot P, Parent A. Dopaminergic neurons intrinsic to the striatum. J. Neurochem. 2007; **101**: 1441–1447.

43 Jankovic J, Chen S, Le WD. The role of Nurr1 in the development of dopaminergic neurons and Parkinson’s disease. Prog. Neurobiol. 2005; **77**: 128–138.

44 Ancín I, Cabranes JA, Vázquez-Álvarez B, Santos JL, Sánchez-Morla E, Alaerts M *et al.* NR4A2: Effects of an orphan receptor on sustained attention in a schizophrenic population. *Schizophr Bull* 2013; **39**: 555–563.
